# Canning Processes Reduce the DNA-Based Traceability of Commercial Tropical Tunas

**DOI:** 10.3390/foods9101372

**Published:** 2020-09-27

**Authors:** Carlo Pecoraro, Valentina Crobe, Alice Ferrari, Federica Piattoni, Anna Sandionigi, Adam J. Andrews, Alessia Cariani, Fausto Tinti

**Affiliations:** 1 Physalia-Courses, 10249 Berlin, Germany; info@physalia-courses.org; 2 Department of Biological, Geological and Environmental Sciences, Alma Mater Studiorum University of Bologna, 48121 Ravenna, Italy; alice.ferrari6@unibo.it (A.F.); federica.piattoni@unibo.it (F.P.); a.andrews@unibo.it (A.J.A.); alessia.cariani@unibo.it (A.C.); fausto.tinti@unibo.it (F.T.); 3 Department of Electronics Information and Bioengineering, Politecnico di Milano, 20133 Milano, Italy; anna.sandionigi@unimib.it

**Keywords:** tropical tunas, DNA barcoding, seafood mislabelling, traceability, species substitution

## Abstract

Canned tuna is one of the most widely traded seafood products internationally and is of growing demand. There is an increasing concern over the vulnerability of canned tuna supply chains to species mislabelling and fraud. Extensive processing conditions in canning operations can lead to the degradation and fragmentation of DNA, complicating product traceability. We here employed a forensically validated DNA barcoding tool (cytochrome b partial sequences) to assess the effects of canning processes on DNA degradation and the identification of four tropical tuna species (yellowfin, bigeye, skipjack and longtail tuna) collected on a global scale, along their commercial chains. Each species was studied under five different canning processes i.e., freezing, defrosting, cooking, and canning in oil and brine, in order to investigate how these affect DNA-based species identification and traceability. The highest percentage of nucleotide substitutions were observed after brine-canning operations and were greatest for yellowfin and skipjack tuna. Overall, we found that DNA degradation significantly increased along the tuna canning process for most specimens. Consequently, most of the specimens canned in oil or brine were misidentified due to the high rate of nucleotide substitution in diagnostic sequences.

## 1. Introduction

The demand for seafood continues to increase worldwide [1]. Some of the most widely traded seafood products are tunas (Perciformes, Scombridae, Thunnini), accounting for ~10% of the international seafood market [2,3]. Tunas are mostly caught by industrial pelagic fisheries, and in 2014 their global landings peaked at 7.7 million metric tons [4]. Catches have since stabilized at around 7.5 million metric tons [1,4]. Four species of tropical tunas dominate global tuna landings: skipjack tuna (*Katsuwonus pelamis*, L. 1758; SKJ), yellowfin tuna (*Thunnus albacares*, Bonnaterre, 1788; YFT), bigeye tuna (*Thunnus obesus*, Lowe, 1839; BET) and longtail tuna (*Thunnus tonggol*, Bleeker, 1851; LOT). Among these, SKJ supports the largest fishery and has the lowest relative market price, while YFT is the second most caught and has the highest market price [5,6]. All four species are used in canning operations, increasing their marketability and transportability. 

Tuna canning consists of several processes that transform fish into preserved products, such as filleting, freezing, defrosting, cooking, and canning in oil or brine. The morphological identification of species becomes impossible after fish filleting. This provides opportunity for the deliberate species substitutions in the commercial tuna market, as reported by several studies [7,8,9]. Seafood mislabelling is a growing and widespread problem with potentially serious economic, environmental and human health impacts [10]. Seafood mislabelling can occur unintentionally or deliberately [11]. Closely related species misidentified at capture and confusion over common names of species used along the supply chain are examples of unintentional substitutions. However, deliberate mislabelling involves the active substitution of species, often for financial gain. This occurs with the substitution of higher value species (e.g., YFT and BET) for lower value species (e.g., SKJ), or when fish from unsustainable or illegal fisheries are used [11]. Due to their high value and great demand, tunas are likely candidates for seafood fraud. Globally, rates of tuna mislabelling vary with different studies reporting a range of values [8,9,12]. However, these studies are difficult to compare because of varying factors such as fishing areas, type of retailer, sampling target and year. Moreover, some species are sold with the generic name “tuna”, further complicating comparisons of mislabelling rates between studies [13].

In the last few years, seafood mislabelling is reported to have decreased in the European Union (EU) commercial area due to the existence of specific labelling regulations (e.g., EU 1379/2013) and the use of appropriate species identification methods [7,14]. The main goal of EU regulations is to provide information to consumers such as commercial and scientific names, thus assuring traceability and identification throughout the value chain [14]. The recent decrease in EU seafood mislabelling rate may be also linked to improvements in traceability testing [9,14]. Among the different species identification for seafood products, DNA barcoding has shown a high reliability at different processing levels [15,16,17,18,19,20]. DNA barcoding represents a powerful tool for rapid species identification based on the amplification and sequencing of short DNA fragments [21]. For studies on animals, the mitochondrial DNA (mtDNA) is generally preferred as genetic detection target over the nuclear DNA because it is inherited without recombination, has a greater abundance (higher copy number) in genomic extracts, and a higher rate of base substitution [22]. A number of studies have shown the applicability of DNA barcoding for accurate species identification of a wide range of tuna products, e.g., canned products, sushi and tuna steaks [23,24,25]. Indeed, DNA is usually more resistant to industrial processes than other molecular markers (e.g., proteins) and can be successfully detected even in small traces [26]. However, canning processes cause the chemical and physical alteration, degradation (as base pair substitutions), and fragmentation of DNA molecules [27]. Due to this, the applicability of molecular methods on canned tuna is still surrounded by uncertainties related mainly to the quantity and quality of extractable DNA [17,19,28,29,30].

**Figure 1:**
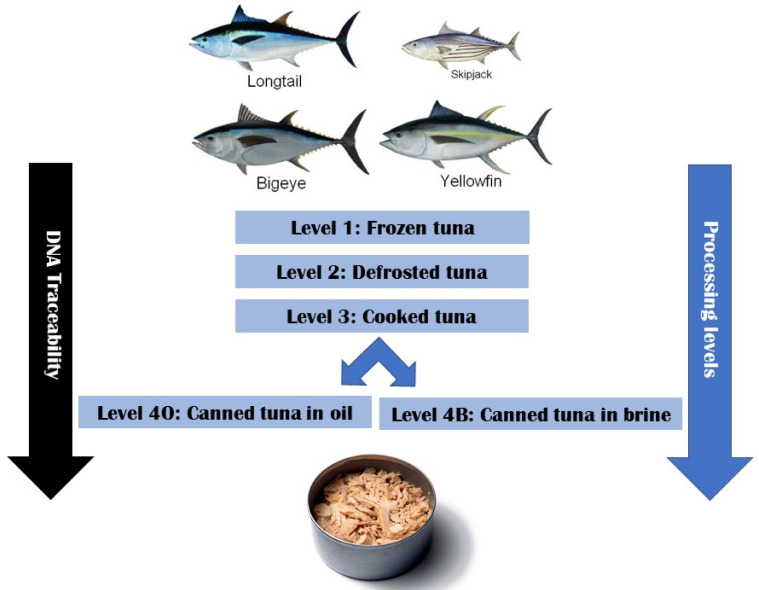
A schematic of the sampling design for the four target tuna species at four processing levels along the tuna canning chain. Each specimen was morphologically identified at the tuna canneries and then genetically identified after having been subjected to each processing level (L1–L4).

Among the different mtDNA markers, the cytochrome b (Cyt b) coding gene shows a relatively high mutation rate. For this reason, Cyt b is powerful for discriminating among closely related tuna species [31]. Botti and Giuffra [32] developed a cocktail of primers that successfully amplifies a Cyt b fragment that allows the discrimination among 17 fish species of the Scombridae family, even in highly degraded samples (i.e., canned tuna and tuna salads). Therefore, in this study we employed this forensically validated DNA barcoding method [32] to assess the impact of DNA degradation on the identification of four target tunas (YFT, BET, SKJ and LOT) along the commercial canned tuna processing chain. This study aimed to: (i) assess the level of DNA degradation, i.e., nucleotide substitution at different processing stages (Figure 1), and (ii) investigate molecular species misidentifications at different processing stages.

## 2. Materials and Methods

### 2.1. Sample Collection

A specific sampling was designed to evaluate the degree of DNA degradation and fragmentation in the different processing steps of tuna canning. Commercial samples of four species: yellowfin tuna (*Thunnus albacares*; YFT), bigeye tuna (*T. obesus*; BET), skipjack tuna (*Katsuwonus pelamis*; SKJ) and longtail tuna (*T. tonggol*, LOT) were obtained from the same tuna canning company on a global scale along with the canning processes (Supplementary Text S1). Tissue samples were collected from the same individuals at each of the processing levels: (L1) frozen; (L2) defrosted; (L3) cooked; (L4O) canned in oil and (L4B) canned in brine (Figure 1, Supplementary Text S1). This sampling design enabled attempts to genetically identify individuals of each species at each processing level. At least three individuals of each species were sampled from each of the FAO Major Fishing Areas where they are caught (http://www.fao.org/fishery/area/search/en) (Table 1). 

### 2.2. DNA Analysis

DNA was extracted from each sample using the General Rapid Easy Extraction System (GREES) DNA Kit for Food (InCura Srl, Italy), following the manufacturer′s instructions. GREES is a spin column method optimized for DNA extraction from food for forensic purposes. Extractions were quality-assessed using 0.8% agarose gel electrophoresis and then stored at −20 °C until further processing. Fragments of the mitochondrial gene Cyt b were amplified via polymerase chain reaction (PCR) following Botti and Giuffra [32]. Three fragments of different lengths (AB: 236 bp, A: 117 bp, and B: 109 bp; Table 1) were amplified due to the expected degradation of DNA in our samples. PCR reactions contained 5 μL of DNA, 375 nM of each primer (forward and reverse), 0.2 mM dNTPs and 0.5 U of HotstartTaq DNA Polymerase (Qiagen S.r.l, Milano, Italy) for a total volume of 25 μL. PCR conditions consisted of 95 °C for 5 min, followed by 40 cycles of: 95 °C for 30 s, 60 °C for 30s and 72 °C for 30 s, followed by a final elongation step at 72 °C for 5 min. PCR products were first evaluated on a 2% agarose gel, and then purified using the ExoSAP-IT™ Express PCR Product Cleanup Reagent (ThermoFisher Scientific Inc., Monza, Italy) following the manufacturer’s instructions. Purified amplicons were sequenced by the Sanger method in an external facility (Macrogen Europe, Amsterdam, The Netherlands). Electropherograms from all successfully amplified samples were retrieved and inspected visually.

### 2.3. DNA Traceability

The DNA sequences obtained were analysed using the software MEGA v7 [33]. Sequences were aligned using the Clustal W algorithm implemented in MEGA v7 and searched against the appropriate nucleotide sequence database in GenBank (www.ncbi.nlm.nih.gov; [34]) using the BLASTn search tool and a full sequence coverage. A sequence similarity of >98% was used as criterion to assess species identifications [35].

In order to evaluate all the diagnostic positions among the four target tuna species, homologous sequences deposited in GenBank were retrieved to build up a sequence reference dataset. A total of 372 sequences (64 of *T. albacares*, 46 of *T. obesus*, 25 of *T. tonggol* and 237 of *K. pelamis*; Supplementary Table S1) were retrieved and aligned to the sequence of the mitochondrial genome of *Thunnus thynnus* L. 1758 (NCBI: NC_004901, positions 14665–14901; see Supplementary Table S1). Sequences obtained from the first processing level (L1, frozen samples) were aligned to the retrieved public sequences and added to the reference dataset.

Automatic Barcode Gap Discovery analysis (ABGD; [36]) was used to estimate the level of intra- and inter-specific variability in the reference dataset. We used the ABGD web-interface available at https://bioinfo.mnhn.fr/abi/public/abgd/abgdweb.html using the default values of relative gap width (X = 1.5) and p-distance.

We estimated the percentage of nucleotide substitutions (NS) of the Cyt b for each species at each processing level (L2–L4) by aligning the obtained sequences to the reference dataset. NS was calculated for each individual sequence as the percentage of the variable positions with respect to the reference level (L1) of the same specimen. The arithmetic average NS was calculated for each level and ocean.

For each level, the genetic species identification of each individual was compared to the morphological identification performed at the tuna canneries. This enabled the inference of how DNA-based traceability is altered along the processing levels of the canned tuna chain for each individual.

### 2.4. Statistical Analysis

All analyses were conducted in R v4.0.0 (R Core Team, 2019). The lme4 package (v1.1-23; [37]) was used to fit Generalized Linear Mixed Models (GLMMs). In order to facilitate reproducible research, we provided the R code used for the models and plots in Supplementary Text S1. The overdisp_fun function, described in Bolker et al. [38], was used to calculate the point estimate of overdispersion in GLMMs with the corresponding significance values. A likelihood ratio test was performed using the *anova()* function.

As the Cyt b sequence is species specific, we modelled NS as a function of consequential canning processes. The following model was fitted, assuming a Poisson distribution:

glmer(nNS ~ Level*Species + (1 | Individual/Species)

where “nNS” = the count of nucleotide substitutions, “Level” = the consequential canning process, “Individual” = the 33 different individuals sampled and “Species” = the morphological identification of each individual/sample.

## 3. Results

### 3.1. DNA Analysis

In total, 165 biological samples of four species were collected on a global scale from October 2016 to February 2017. We obtained 60 samples each of YFT and SKJ from the Atlantic, Indian, Western-Central Pacific and Eastern Pacific Oceans. A total of 30 samples of BET were obtained from the Indian and Eastern Pacific Oceans, and 15 samples of LOT from the Indian Ocean (Table 1). Sampling of each species was restricted to the ocean basins where capture and processing takes place.

At the processing levels 1, 2 and 3, the longest fragment (AB) was successfully amplified except for a few level 3 samples in which only the amplification of the fragment A was obtained due to DNA degradation. At the level 4O, only the short B fragments successfully amplified due to a high level of DNA degradation (Table 1). Two SKJ specimens for the levels 3 and 4B and one YFT level L3 sample failed to amplify

### 3.2. DNA Traceability

Ninety DNA samples from the levels 1, 2 and 3 were successfully amplified and sequenced using the longest fragment (AB) with a final alignment length of 236 bp (Supplementary Table S2). Molecular identifications using BLASTn matched morphological identifications for 86 out of 90 (95.55%) AB fragments, the exceptions being four samples at levels 2 and 3. Eight sequences could only be amplified and sequenced using the fragment A, with a final alignment length of 117 bp (Supplementary Table S2). Of these, seven samples were from level 3 and one sample was from level 4O (Table 1). A further 64 samples could only be amplified and sequenced using fragment B, with a final alignment length of 109 bp. Molecular identifications matched morphological identifications for only 34 out of 64 (53.12%) B fragments, with varying success between species (Supplementary Table S3).

The ABGD analysis (Supplementary Figure S1) revealed an intra-specific nucleotide sequence variation for all species with a pairwise p-distance values range from 0 to 0.05. This variation was markedly different from inter-specific variation values (range: 0.09–0.14), presenting a clear barcode gap in the reference Cyt b sequence dataset. This allowed the use of the Cyt b sequence marker to correctly assess specific assignment regardless of geographical/individual intra-specific variability.

**Figure 2:**
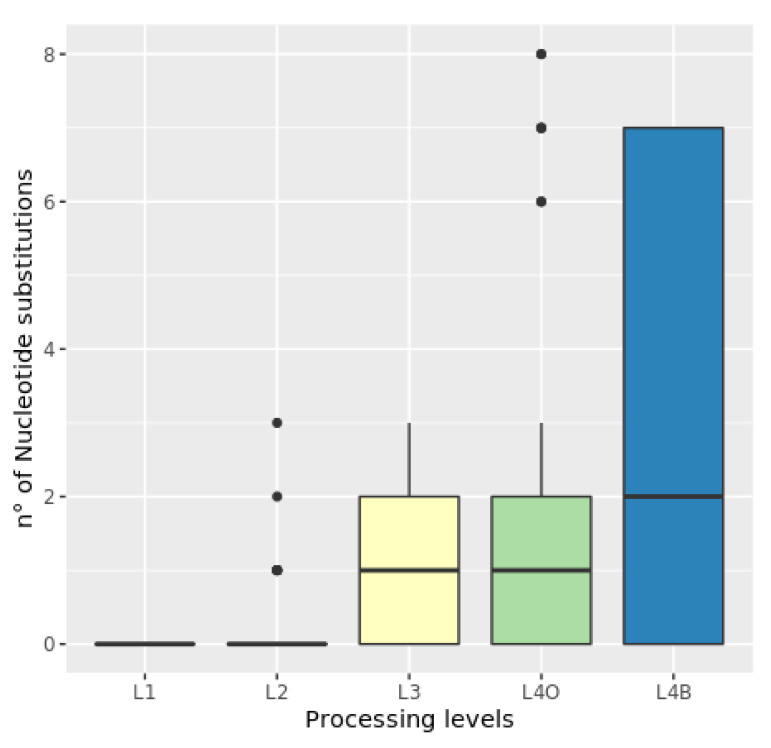
Box plot of nucleotide substitutions at the four processing levels (L1–L4).

**Figure 3:**
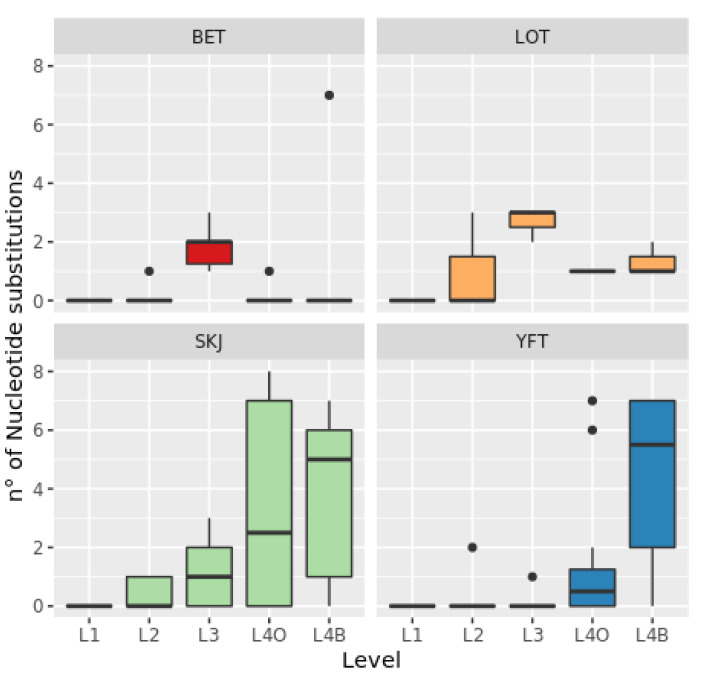
Box plots of nucleotide substitution counts at the four processing levels (L1–L4) and for the four target species: bigeye tuna (BET), longtail tuna (LOT), skipjack tuna (SKJ) and yellowfin tuna (YFT).

Nucleotide substitutions increased through levels 1 to 4 with the highest value at level 4B (Figure 2). There was a significant model effect when processing level was considered in interaction with species (χ^2^ (12) = 60.549, *p* < 0.00001). Our results show a greater number of NS for SKJ and YFT compared to the other species, especially at the levels 4O and 4B (Figure 3). We did not observe a significant effect of the sampling area on NS for any of the species or levels (see Supplementary Text S1).

#### 3.2.1. YFT

We observed a high percentage of NS for YFT at level 4O in Atlantic and Indian Ocean samples, with averages of 3.06% and 2.14%, respectively, and at level 4B which ranged from 2.75% to 6.42% (Figure 3). At levels 4O and 4B, NS occurred at seven diagnostic nucleotide positions that hindered the discrimination between our four target species. A greater percentage of NS was observed in YFT samples from the Atlantic Ocean at level 4O (3.06%) and 4B (6.42%). On the contrary, no NS occurred in the 4O samples from the Eastern Pacific Ocean and were rarely observed in the Western-Central Pacific Ocean (only one sample, 0.31%). A lower percentage of NS at level 4B was observed for Indian Ocean samples (2.75%). Comparative analysis between the different geographic areas identified a mutation (C substituted for T) at position 144 in all Eastern Pacific Ocean samples. Most YFT samples at levels 4O and 4B that presented a substitution in one of the seven diagnostic nucleotide positions were genetically misidentified as SKJ, and one Atlantic Ocean sample (level 4O) was misidentified as BET (Table 2). Four sequences at level 4B displayed similarity scores of <98% when compared with public sequences, thus preventing robust molecular identification. Only one sample at level 2 and one at level 3, both from the Atlantic Ocean, displayed very low percentages of NS (0.28% and 0.21%, respectively), without affecting the correct molecular identification of the samples.

#### 3.2.2. BET

BET samples were obtained only from the Indian and Eastern Pacific Oceans. We observed a clear geographic pattern in the degree of NS in BET (Figure 3). A greater percentage of NS was observed at level 4O (0.31%) and 4B (2.14%) in the Eastern Pacific Ocean than in the Indian Ocean (0%). Samples from both areas displayed NS for the level 3 (EPO: 1.99%; IO: 1.14%), but the effects of the sampling area on the percentage of NS was not significant (*p* > 0.05). Despite our observations of NS, only one BET individual was misidentified as SKJ, at level 4B (Table 2).

#### 3.2.3. SKJ

Nucleotide substitutions for SKJ significantly increased between levels 2 and 4, with variations among areas. The greatest percentages of NS were observed in samples from the Eastern Pacific Ocean, at level 3 (1.84%). Indian Ocean samples displayed a high percentage of NS at level 4O (4.89%) greater than in any other area. High percentages were also observed at the level 4B (4.89%) and in the Western-Central Pacific (4.28%), Eastern Pacific (3.98%), and Indian Oceans (1.83%). At levels 4O and 4B, NS occurred mainly in diagnostic positions (from position 63 to 213 in the alignment), which discriminate between SKJ and the three tuna species studied herein. For this reason, most SKJ at these levels were misidentified as BET or for two specimens similarity scores were too low for robust identifications (<98%; Table 2). All SKJ from levels 2 and 3 matched morphological identifications, except one individual from the Eastern Pacific Ocean at level 3 which shared <98% of sequence similarity with public sequences.

#### 3.2.4. LOT

LOT samples were obtained only from the Indian Ocean. Due to the low number of samples obtained (*n* = 15), the assessment of the NS in LOT samples is limited. NS were observed at all levels, with the greatest percentage (1.42%) at level 3, followed by level 4B (1.22%). NS at all levels occurred in diagnostic positions, which discriminate LOT from the other species studied herein. Indeed, three LOT specimens at levels 4O and 4B were genetically misidentified as BET, while three specimens at levels 2 and 3 were genetically misidentified as *T. thynnus* or YFT with the same value of sequence similarity (Table 2).

## 4. Discussion and Conclusions

The use of DNA barcoding may be limited in highly processed tuna products where DNA could be degraded into smaller fragments that are difficult to amplify [39]. However, Polymerase Chain Reaction-Forensically Informative Sequencing (PCR-FINS) and mini-DNA barcoding methods were successfully applied for species identification in highly processed meat foods [40] and in museum specimens [41,42].

Our results show a significant increase in the NS percentage between defrosting (L2) to canning (L4, in oil or brine) as processing became more intensive. This is most probably to be related to the high temperatures and chemical/physical treatments used during the canning process, which are likely to negatively affect the quality and quantity of DNA by fragmenting DNA molecules [43]. Interestingly, NS were significantly higher after brine canning (level 4B) than after oiling (level 4O). This suggests that the type of preservative employed in canning not only influences quantity of the DNA extracted [28], but its quality and therefore its traceability.

The observed increase in NS percentage was more evident in yellowfin and skipjack tuna samples, which had the largest sample sizes (YFT = 59 and SKJ = 58). Therefore, the sample size appears to affect this trend due to the large degrees of variation observed. However, the other two species with lower sample sizes presented contrasting patterns of NS: BET (*n* = 30) displayed 1.57% NS at level 3, a decrease at level 4O and an increase at level 4B, while LOT (*n* = 15) displayed unexpectedly the greatest degree of NS at level 3.

For YFT, Cyt b barcoding of defrosted and cooked tuna confirmed the morphological species identification of all individuals at the canneries. However, due to the NS occurring at some of the seven diagnostic positions of the 236-bp alignment, three YFT individuals canned in oil and ten individuals canned in brine were genetically misidentified. SKJ exhibited similar results to those obtained for YFT. Indeed, the molecular and morphological results matched the identification of all defrosted and cooked SKJ, except one specimen for the levels 2 and 3. Most SKJ canned in oil or brine were misidentified as BET, due to the high rate of NS in discriminant positions. Conversely, in the canned BET, even if NS occurred, only one sample was misidentified as SKJ. For BET samples, misidentification was rarer than in other species because NS did not occur in diagnostic positions [44]. This contrasts with LOT samples, in which a large number of NS at most of the diagnostic positions impacted species identification of defrosted and cooked individuals.

Given the very high percentage of specimens correctly-identified at the species level with the fragment AB and the relative high percentage with the A and B fragments, our results confirmed that tropical tunas can be reliably identified with Cyt b barcodes with high BLAST sequence similarities, even after commercial processing. It is important to note that in samples with highly degraded DNA, as we have here from cooked and canned tuna (specimens from levels 3 and 4), sequences should not be entered into GenBank to avoid low-quality references and thus misidentification in future studies [45]. The Cyt b primer cocktail developed by Botti and Giuffra [32] represents an efficient molecular tool to identify tuna products and to validate contents label information for the four species studied herein. This set of primers allowed the PCR amplification and sequencing of both longer (236 bp) and shorter (117 bp and 109 bp) fragments of the mitochondrial gene Cyt b, obtaining a unique and clearly distinguishable sequence for each of the four species. Our results, coupled with the reliability of the Cyt b gene for the identification of tropical tuna species paired and the monitoring of traceable reference individuals along the processing chain (i.e., with no species substitution from one level to the other) led us to consider that species mislabelling detected in canned tunas could be related to the loss of traceability (nucleotide substitution), due to DNA degradation and fragmentation and not to an intentional mislabelling. Although it is well established that extensive processing conditions in canning operations can lead to the degradation and fragmentation of DNA samples [22,46], the majority of previous studies on tunas were focused on the highly processed final products (i.e., canned tuna, sushi and sashimi). These studies reported high levels of mislabelling in tuna products [13] but none of them investigated how genetic traceability is affected by the canning treatments. Instead, here we monitored along with the canning production process, the reduction of DNA traceability of tuna products associated to the DNA degradation and fragmentation that can occur during the processing steps. Other studies present lower levels of mislabelling when considering different factors [9]. For instance, Pardo et al. [12] reported that 18% of tuna products were mislabelled, Gordoa et al. [8] observed 37% in fresh and frozen tuna in Spain at the point of sale, and 48% in restaurants, and Sotelo et al. [9] found that 6.7% of fresh and frozen tuna were mislabelled compared to 7.8% for canned products. Our results indicate that the DNA of our specimens was highly degraded and fragmented, and that all canned specimens, except those of the BET, lost their diagnostic nucleotide features resulting in taxonomic misidentification. In these cases, suggestions of mislabelling would be erroneous as the misidentification observed was a consequence of the NS related to the degradation and fragmentation of the DNA caused by canning operations and indeed future studies must consider this important point.

We conclude that while our sampling approach is difficult to replicate, this approach was much needed to validate the impact of tuna canning processes on traceability. In previous studies, authors did not have the possibility to compare the results obtained from different processing levels, and this stresses the key challenges of global tuna sustainability and traceability. Firstly, tropical tuna products rely on catches from oceanic waters, posing logistical challenges such as knowing the geographic location where a fish was caught due to illegal, unregulated and unreported fishing. Secondly, in the monitoring of fraud and species substitutions, it is crucially important that morphological identifications are made at the canneries. However, it is extremely difficult to control this step. Taking these lessons into account, closer collaborations are needed between researchers and the tuna industry, especially in geographic areas exporting high volumes of tuna products to Europe. Our results clearly show how DNA barcoding of highly processed products can lead to perceived species misidentifications, which can fuel the lack of transparency that prevents the consumers from environmentally conscious purchasing decisions. Global tropical tuna sustainability and traceability can therefore only be achieved through robust international collaborations and coordinated governance efforts among processors, traders, importers, transporters, marketers and researchers in order to trace tuna products from ocean to plate.

## Figures and Tables

**Table 1:** Sampling and sequencing information for each specimen of the four species studied herein (Yellowfin tuna: YFT; Bigeye tuna: BET; Longtail tuna; LOT, Skipjack tuna: SKJ). The four processing levels: L1-L4. Amplified fragment of the mitochondrial gene Cyt b that were sequenced: AB (236 bp), A (117 bp), B (109 bp). Amplification failures: “na“. FAO Major Fishing Areas are detailed online: http://www.fao.org/fishery/area/search/en.

Species	FAO Major Fishing Area	Specimen	Fork Length (cm)	Total Weight (kg)	Amplified fragment (AB, A, B)				
					L1	L2	L3	L4O	L4B
**YFT**	Atlantic Ocean -FAO 34	1	104–140	51	AB	AB	AB	B	B
		2	101–168	44	AB	AB	AB	B	B
		3	107–138	52	AB	AB	na	B	B
	Indian Ocean -FAO 51	1	54.5	3.06	AB	AB	AB	B	B
		2	57	3.6	AB	AB	AB	B	B
		3	61.5	4.28	AB	AB	AB	B	B
	Eastern Pacific Ocean -FAO 87	1	55.88	3.37	AB	AB	AB	B	B
		2	54.61	3.16	AB	AB	AB	B	B
		3	55.88	3.69	AB	AB	AB	B	B
	Western-Central Pacific Ocean -FAO 71	1	45	1.7	AB	AB	AB	B	B
		2	48.5	2.3	AB	AB	AB	B	B
		3	41	1.2	AB	AB	AB	B	B
**BET**	Indian Ocean -FAO 51	1	41	1.6	AB	AB	A	B	B
		2	45	1.7	AB	AB	A	B	B
		3	42	1.7	AB	AB	A	B	B
	Eastern Pacific Ocean -FAO 87	1	71.12	7.47	AB	AB	A	B	B
		2	74.93	8.35	AB	AB	A	B	B
		3	68.58	6.37	AB	AB	A	B	B
**LOT**	Indian Ocean -FAO 51	1	42	1	AB	AB	A	B	B
		2	48	1.6	AB	AB	AB	B	B
		3	44	1.2	AB	AB	AB	B	B
**SKJ**	Atlantic Ocean -FAO 34	1	41–60	4.95	AB	AB	AB	A	B
		2	42–63.5	5.45	AB	AB	AB	B	B
		3	42–62	5.3	AB	AB	na	B	B
	Indian Ocean -FAO 51	1	48	3.54	AB	AB	AB	B	B
		2	47	3.48	AB	AB	AB	B	na
		3	52	4.26	AB	AB	AB	B	B
	Eastern Pacific Ocean -FAO 87	1	64.77	5.22	AB	AB	AB	B	B
		2	59.69	4.97	AB	AB	AB	B	B
		3	58.42	4.11	AB	AB	AB	B	B
	Western-Central Pacific Ocean -FAO 71	1	48	2.1	AB	AB	AB	B	B
		2	46	1.8	AB	AB	AB	B	B
		3	45	1.9	AB	AB	AB	B	B

**Table 2:** Samples that were genetically misidentified at each processing level or had similarity scores <98% (*). Yellowfin tuna: YFT, Bigeye tuna: BET, Skipjack tuna: SKJ, Longtail tuna: LOT. Atlantic Ocean: AO, Indian Ocean: IO, Western-Central Pacific Ocean: WCPO, Eastern Pacific Ocean: EPO.

Level	Species	Ocean	Sample ID	Fragment	BLAST Identification	Similarity (%)	Accession Number
L2	LOT	IO	LOT-IO-L2-3	AB	*T. thynnus*, *T. albacares*	98.73%	MG017705.1, MG017687.1
L3	LOT	IO	LOT-IO-L3-2	AB	*T. thynnus*, *T. albacares*	98.73%	MG017705.1, MG017687.1
L3	LOT	IO	LOT-IO-L3-3	AB	*T. thynnus*, *T. albacares*	98.73%	MG017705.1, MG017687.1
L3	SKJ	EPO	SKJ-EPO-L3-2	AB	*K. pelamis*	96.19% *	KP669130.1
L4O	LOT	IO	LOT-IO-L4O-1	B	*T. obesus*	98.17%	MG017696.1
L4O	YFT	AO	YFT-AO-L4O-1	B	*T. obesus*	98.17%	MG017696.1
L4O	YFT	AO	YFT-AO-L4O-2	B	*K. pelamis*	100%	KP669132.1
L4O	YFT	IO	YFT-IO-L4O-1	B	*K. pelamis*	98.17%	KP669172.1
L4O	SKJ	AO	SKJ-AO-L4O-2	B	*K. pelamis*	97.25% *	KP669132.1
L4O	SKJ	EPO	SKJ-EPO-L4O-1	B	*T. obesus*	98.17%	MG017696.1
L4O	SKJ	EPO	SKJ-EPO-L4O-2	B	*T. obesus*	98.17%	MG017696.1
L4O	SKJ	IO	SKJ-IO-L4O-1	B	*T. obesus*	98.17%	MG017696.1
L4O	SKJ	IO	SKJ-IO-L4O-2	B	*T. obesus*	98.17%	MG017696.1
L4O	SKJ	WCPO	SKJ-WCPO-L4O-1	B	*T. obesus*	98.17%	MG017696.1
L4B	BET	EPO	BET-EPO-L4B-3	B	*K. pelamis*	100%	AB098093.1
L4B	LOT	IO	LOT-IO- L4B-1	B	*T. obesus*	98.17%	MG017696.1
L4B	LOT	IO	LOT-IO- L4B-2	B	*T. obesus*	98.17%	MG017696.1
L4B	YFT	AO	YFT-AO-L4B-1	B	*K. pelamis*	100%	KP669132.1
L4B	YFT	AO	YFT-AO-L4B-2	B	*K. pelamis*	100%	KP669132.1
L4B	YFT	AO	YFT-AO-L4B-3	B	*K. pelamis*	100%	KP669132.1
L4B	YFT	EPO	YFT-EPO- L4B-1	B	*K. pelamis*, *T. albacares, T. obesus*	96.33%	KP669132.1, MG017683.1, KJ018958.1
L4B	YFT	EPO	YFT-EPO-L4B-3	B	*T. albacares*	97.25% *	MG017683.1
L4B	YFT	EPO	YFT-EPO-L4B-2	B	*K. pelamis*	99.08%	KP669132.1
L4B	YFT	IO	YFT-IO-L4B-2	B	*T. albacares*, *T.obesus*	97.25%	MG017683.1, MG017696.1
L4B	YFT	IO	YFT-IO-L4B-3	B	*K. pelamis*	99.08%	KP669172.1
L4B	YFT	WCPO	YFT-WCPO-L4B-1	B	*K. pelamis*	99.08%	KP669132.1
L4B	YFT	WCPO	YFT-WCPO- L4B-3	B	*T. albacares*, *T.obesus*	97.25%	MG017683.1, KJ018958.1
L4B	SKJ	AO	SKJ-AO-L4B-1	B	*T. obesus*	98.17%	MG017696.1
L4B	SKJ	AO	SKJ-AO-L4B-2	B	*T. obesus*	98.17%	MG017696.1
L4B	SKJ	EPO	SKJ-EPO-L4B-1	B	*T. obesus*	98.17%	MG017696.1
L4B	SKJ	EPO	SKJ-EPO-L4B-2	B	*T. obesus*	98.17%	MG017696.1
L4B	SKJ	IO	SKJ-IO-L4B-1	B	*K. pelamis*	97.25% *	KP669132.1
L4B	SKJ	WCPO	SKJ-WCPO-L4B-1	B	*T. obesus*	98.17%	MG017696.1
L4B	SKJ	WCPO	SKJ-WCPO-L4B-2	B	*T. obesus*	98.17%	MG017696.1

## Supplementary Materials

The following are available online at https://www.mdpi.com/2304-8158/9/10/1372/s1. Text S1: Description of the canning processes and tissue sampling. Complete list of R code for all models and plots used in this study. Table S1: 35 diagnostic positions of the Cyt b sequences of the four target species (YFT, yellowfin tuna, *Thunnus albacares*; BET, bigeye tuna, *Thunnus obesus*; SKJ, skipjack tuna, *Katsuwonus pelamis*; LOT, longtail tuna, *Thunnus tonggol*) aligned against to the sequence of the mitochondrial genome of *Thunnus thynnus* (NCBI: NC_004901, positions 14665–14901, 236 bp) and to public available sequences for each of the four tuna species used as reference. The sequences obtained from the first processing level (L1-frozen samples) were aligned to the public sequences and they were considered as reference nucleotide substitutions = 0. Table S2: Nucleotide substitution within the Cyt b sequences of the four target species (YFT, yellowfin tuna, *Thunnus albacares*; BET, bigeye tuna, *Thunnus obesus*; SKJ, skipjack tuna, *Katsuwonus pelamis;* LOT, longtail tuna, *Thunnus tonggol*) aligned against to the sequence of the mitochondrial genome of *Thunnus thynnus* (NCBI: NC_004901, positions 14665–14901, 236 bp). Sequences from other levels (L2–L4) were compared with the reference level (L1). Nucleotide substitution was calculated for each specimen as a percentage of the variable positions. Table S3: Summary information of the samples used in this study and their molecular identification results. Morph. ID = Morphological identification for the four species (*Thunnus albacares* = YFT; *Thunnus obesus* = BET; *Katsuwonus pelamis* = SKJ; *Thunnus tonggol* = LOT;) achieved at the canneries; Ocean (Indian Ocean = IO; Atlantic Ocean = AO; Western-Central Pacific Ocean = WCPO; Eastern Pacific Ocean = EPO); amplified fragment of the of the mitochondrial gene Cyt b (AB, A, B); Blast ID = species genetically identified in GenBank (www.ncbi.nlm.nih.gov) using the BLASTn search tool;% Similarity = genetic similarity in GenBank; Accession number of the species genetically identified in GenBank. Figure S1: Output of the Automatic Barcode Gap Discovery (ABGD) web-interface of the Cyt b sequences reference dataset of 236 bp calculated with p-distance. A—Histogram of distances. B—Ranked distances.

## References

[1] FAO (2018). The State of World Fisheries and Aquaculture 2018—Meeting the Sustainable Development Goals.

[2] Guillotreau P., Squires D., Sun J., Compeán G.A. (2016). Local, regional and global markets: What drives the tuna fisheries?. Rev. Fish. Biol. Fisher..

[3] Brill R.W., Hobday A.J. (2017). Tunas and their fisheries: Safeguarding sustainability in the twenty-first century. Rev. Fish. Biol. Fisher..

[4] FAO (2016). The State of World Fisheries and Aquaculture 2016—Contributing to Food Security and Nutrition for All.

[5] Thai Union Group Public Company Limited Annual Report 2017. https://investor.thaiunion.com/misc/ar/20180329-tu-ar2017-en.pdf.

[6] Pecoraro C., Zudaire I., Bodin N., Murua H., Taconet P., Díaz-Jaimes P., Cariani A., Tinti F., Chassot E. (2017). Putting all the pieces together: Integrating current knowledge of the biology, ecology, fisheries status, stock structure and management of yellowfin tuna (*Thunnus albacares*). Rev. Fish. Biol. Fisher.

[7] Bénard-Capelle J., Guillonneau V., Nouvian C., Fournier N., Loët K.L., Dettai A. (2015). Fish mislabelling in France: Substitution rates and retail types. PeerJ.

[8] Gordoa A., Carreras G., Sanz N., Viñas J. (2017). Tuna species substitution in the Spanish commercial chain: A knock-on effect. PLoS ONE.

[9] Sotelo C.G., Velasco A., Perez-Martin R.I., Kappel K., Schröder U., Verrez-Bagnis V., Jérôme M., Mendes R., Silva H., Mariani S. (2018). Tuna labels matter in Europe: Mislabelling rates in different tuna products. PLoS ONE.

[10] Jacquet J., Pauly D. (2008). Funding Priorities: Big Barriers to Small-Scale Fisheries. Conserv. Biol..

[11] Barendse J., Roel A., Longo C., Andriessen L., Webster L.M.I., Ogden R., Neat F. (2019). DNA barcoding validates species labelling of certified seafood. Curr. Biol..

[12] Pardo M.Á., Jiménez E., Pérez-Villarreal B. (2016). Misdescription incidents in seafood sector. Food Control.

[13] Willette D.A., Simmonds S.E., Cheng S.H., Esteves S., Kane T.L., Nuetzel H., Pilaud N., Rachmawati R., Barber P.H. (2017). Using DNA barcoding to track seafood mislabeling in Los Angeles restaurants. Conserv. Biol..

[14] Mariani S., Griffiths A.M., Velasco A., Kappel K., Jérôme M., Perez-Martin R.I., Schröder U., Verrez-Bagnis V., Silva H., Vandamme S.G. (2015). Low mislabeling rates indicate marked improvements in European seafood market operations. Front. Ecol. Environ..

[15] Lenstra J.A. (2013). DNA methods for identifying plant and animal species in food. Food Authenticity and Traceability.

[16] Ward R.D., Zemlak T.S., Innes B.H., Last P.R., Hebert P.D.N. (2005). DNA barcoding Australia’s fish species. Philos. Soc. B.

[17] Rasmussen R.S., Morrissey M.T. (2008). DNA-Based methods for the identification of commercial fish and seafood species. Compr. Rev. Food Sci. F.

[18] Viñas J., Tudela S. (2009). A validated methodology for genetic identification of tuna species (Genus *Thunnus*). PLoS ONE.

[19] Hellberg R.S.R., Morrissey M.T. (2011). Advances in DNA-based techniques for the detection of seafood species substitution on the commercial market. JALA J. Assoc. Lab. Autom..

[20] Mata W., Chanmalee T., Punyasuk N., Thitamadee S. (2020). Simple PCR-RFLP detection method for genus- and species-authentication of four types of tuna used in canned tuna industry. Food Control.

[21] Hebert P.D.N., Cywinska A., Ball S.L., deWaard J.R. (2003). Biological identifications through DNA barcodes. Ser. B Biol..

[22] Pardo M.A., Pérez-Villareal B. (2004). Identification of commercial canned tuna species by restriction site analysis of mitochondrial DNA products obtained by nested primer PCR. Food Chem..

[23] Shokralla S., Hellberg R.S., Handy S.M., King I., Hajibabaei M. (2015). A DNA mini-barcoding system for authentication of processed fish products. Sci. Rep. UK.

[24] Nagalakshmi K., Annam P.K., Venkateshwarlu G., Pathakota G.B., Lakra W.S. (2016). Mislabeling in Indian seafood: An investigation using DNA barcoding. Food Control.

[25] Tinacci L., Stratev D., Vashin I., Chiavaccini I., Susini F., Guidi A., Armani A. (2018). Seafood labelling compliance with European legislation and species identification by DNA barcoding: A first survey on the Bulgarian market. Food Control.

[26] Bottero M.T., Dalmasso A., Cappelletti M., Secchi C., Civera T. (2007). Differentiation of five tuna species by a multiplex primer-extension assay. J. Biotechnol..

[27] Bauer T., Weller P., Hammes W.P., Hertel C. (2003). The effect of processing parameters on DNA degradation in food. Eur. Food Res. Technol..

[28] Chapela M.J., Sotelo C.G., Pérez-Martín R.I., Pardo M.Á., Pérez-Villareal B., Gilardi P., Riese J. (2007). Comparison of DNA extraction methods from muscle of canned tuna for species identification. Food Control.

[29] Pollack S.J., Kawalek M.D., Williams-Hill D.M., Hellberg R.S. (2018). Evaluation of DNA barcoding methodologies for the identification of fish species in cooked products. Food Control.

[30] Pafundo S., Agrimonti C., Maestri E., Marmiroli N. (2007). Applicability of SCAR markers to food genomics:  olive oil traceability. J. Agric. Food Chem..

[31] Abdullah A., Rehbein H. (2015). The differentiation of tuna (family: Scombridae) products through the PCR-based analysis of the cytochrome b gene and parvalbumin introns. J. Sci. Food Agr..

[32] Botti S., Giuffra E. (2010). Oligonucleotide indexing of DNA barcodes: Identification of tuna and other scombrid species in food products. BMC Biotechnol..

[33] Kumar S., Stecher G., Tamura K. (2016). MEGA7: Molecular Evolutionary Genetics Analysis Version 7.0 for bigger datasets. Mol. Biol. Evol..

[34] Sayers E.W., Cavanaugh M., Clark K., Ostell J., Pruitt K.D., Karsch-Mizrachi I. (2020). GenBank. Nucleic Acids Res..

[35] Barbuto M., Galimberti A., Ferri E., Labra M., Malandra R., Galli P., Casiraghi M. (2010). DNA barcoding reveals fraudulent substitutions in shark seafood products: The Italian case of “palombo” (*Mustelus* spp.). Food Res. Int..

[36] Puillandre N., Lambert A., Brouillet S., Achaz G. (2012). ABGD, Automatic Barcode Gap Discovery for primary species delimitation. Mol. Ecol..

[37] Bates D., Mächler M., Bolker B., Walker S. (2015). Fitting linear mixed-effects models using lme4. J. Stat. Softw..

[38] Bolker B.M., Brooks M.E., Clark C.J., Geange S.W., Poulsen J.R., Stevens M.H.H., White J.-S.S. (2009). Generalized linear mixed models: A practical guide for ecology and evolution. Trends Ecol. Evol..

[39] Krčmář P., Piskatá Z., Servusová E. (2019). Identification of tuna species *Thunnus albacares* and *Katsuwonus pelamis* in canned products by real-time PCR method. Acta Vet. Brno..

[40] Soman M., Paul R.J., Antony M., Padinjarattath Sasidharan S. (2020). Detecting mislabelling in meat products using PCR–FINS. J. Food Sci. Technol..

[41] Hajibabaei M., Smith M.A., Janzen D.H., Rodriguez J.J., Whitfield J.B., Hebert P.D.N. (2006). A minimalist barcode can identify a specimen whose DNA is degraded. Mol. Ecol. Notes.

[42] Leone A., Puncher G.N., Ferretti F., Sperone E., Tripepi S., Micarelli P., Gambarelli A., Sarà M., Arculeo M., Doria G. (2020). Pliocene colonization of the Mediterranean by Great White Shark inferred from fossil records, historical jaws, phylogeographic and divergence time analyses. J. Biogeogr..

[43] Peano C., Samson M.C., Palmieri L., Gulli M., Marmiroli N. (2004). Qualitative and quantitative evaluation of the genomic DNA extracted from GMO and non-GMO foodstuffs with four different extraction methods. J. Agric. Food Chem..

[44] Higashi R., Sakuma K., Chiba S.N., Suzuki N., Chow S., Semba Y., Okamoto H., Nohara K. (2016). Species and lineage identification for yellowfin *Thunnus albacares* and bigeye *T. obesus* tunas using two independent multiplex PCR assays. Fish. Sci..

[45] Quinteiro J., Sotelo C.G., Pérez-martín R.I., Rey-méndez M. (1998). Use of mtDNA direct Polymerase Chain Reaction (PCR) sequencing and PCR-Restriction fragment length polymorphism methodologies in species identification of canned tuna. J. Agric. Food Chem..

[46] Lowenstein J.H., Amato G., Kolokotronis S.-O. (2009). The real *maccoyii*: Identifying tuna sushi with DNA barcodes—Contrasting characteristic attributes and genetic distances. PLoS ONE.

