# Supplementary material for: Canning Processes Reduce the DNA-Based Traceability of Commercial Tropical Tunas
Source: Foods. 2020 Sep 27;9(10):1372. doi: 10.3390/foods9101372 (PMC7650566; doi:10.3390/foods9101372)
Supplement: Supplementary file 1 [file foods-09-01372-s001.zip › SUPPLEMENTARY/Supplementary Text S1_foods-943909_rev18_09_2020.docx]

**Pecoraro et al. Canning processes reduce the DNA- based traceability of commercial tropical tunas**

**Supplementary Methods**

*Canning processing levels and tissue sampling*

Tunas were preserved onboard in wells with brine freezing at a temperature of -20° C. At the cannery, frozen individuals were thawed by means of running water at a temperature of 10-15 °C. Once defrosted, each fish was morphologically identified by trained personnel. For the first two processing levels (L1 and L2, Figure 1), a portion of skeletal muscle or finclip was excised from each fish and immediately stored in 96% molecular grade ethanol. Then, each fish was cooked by heating at a temperature in the range of 102 ºC to 104 ºC for the time necessary to make it possible to hand pick the light meat from the carcass and also to remove some of the oil from oily fish. The pre-cooking time for individual batches can vary widely according to the size of tuna (ranging from 1 h for small-sized individuals to 8 h for large-sized individuals). For the processing level L3 (Figure 1) a portion of cooked white muscle was excised and immediately stored in 96% molecular grade ethanol. Tunas were then cut in loins and filled into cans by tuna filler machines (processing level L4, Figure 1), where additives such brine (4-B) or oil (4-O) were added. All the sampling procedures to collect individual specimens were carried out using disposable materials and cleaning equipment to prevent the risk of cross species contamination.

*R-codes*

R code for all models and plots are given in the RMarkdown Supplementary file R_data_food_Pecoraro_et_al.Rmd.
