# Supplementary material for: Canning Processes Reduce the DNA-Based Traceability of Commercial Tropical Tunas
Source: Foods. 2020 Sep 27;9(10):1372. doi: 10.3390/foods9101372 (PMC7650566; doi:10.3390/foods9101372)
Supplement: Supplementary file 1 [file foods-09-01372-s001.zip › SUPPLEMENTARY/Table S1_foods-943909_rev18_09_2020.pdf]

Supplementary Material

Pecoraro et al. Canning processes reduce DNA-based traceability of commercial tropical tunas

Table S1: 35 diagnostic positions of the Cyt b sequences of the four target species (YFT, yellowfin tuna, *Thunnus albacares* ; BET, bigeye tuna, *Thunnus obesus* ; SKJ, skipjack tuna, *Katsuwonus pelamis* ; LOT, longtail tuna, *Thunnus tonggol*) aligned against to the ortholog sequence of the mitochondrial genome of *Thunnus thynnus* (NCBI: NC_004901, positions 14665 – 14901, 236 bp) and to publicly available sequences for each of the four tuna species used as reference. The sequences obtained from the first processing level (L1-frozen samples) were aligned to the public sequences and they were considered as reference NS=0.

		2	6	9	18	21	30	33	36	39	45	63	69	72	75	84	93	96	102	105	111	120	123	129	132	144	156	162	174	180	183	192	198	213	219	231
NC 004901	*Thunnus thynnus*	T	C	C	A	T	C	T	C	A	A	A	A	C	G	T	C	C	T	C	C	A	A	G	T	T	T	C	C	T	C	T	T	A	T	T
DQ080271	*Thunnus albacares*	.	.	.	.	.	.	.	.	.	G	.	.	.	A	.	.	.	.	.	.	.	.	.	.	.	.	T	.	.	T	.	.	.	.	.
DQ080281	*Thunnus albacares*	.	.	.	.	.	.	.	.	.	G	.	.	.	A	.	.	.	.	.	.	.	.	.	.	.	.	T	.	.	T	.	.	.	.	.
DQ080282	*Thunnus albacares*	.	.	.	.	.	.	.	.	.	G	.	.	.	A	.	.	.	.	.	.	.	.	.	.	.	.	T	.	.	T	.	.	.	.	.
DQ080283	*Thunnus albacares*	.	.	.	.	.	.	C	.	.	G	.	.	.	A	.	.	.	C	.	.	.	.	.	.	.	.	T	.	.	.	C	.	.	.	.
DQ080284	*Thunnus albacares*	.	.	.	.	.	.	C	.	.	G	.	.	.	A	.	.	.	.	.	.	.	.	.	.	.	.	T	.	.	T	.	.	.	.	.
DQ080285	*Thunnus albacares*	.	.	.	.	.	.	.	.	.	G	.	.	.	A	.	.	.	.	.	.	.	.	.	.	.	.	T	.	.	T	.	.	.	C	.
DQ080286	*Thunnus albacares*	.	.	.	.	.	.	.	.	.	G	.	.	.	A	.	.	.	.	.	.	.	.	.	.	.	.	T	.	.	T	.	.	.	.	.
DQ080287	*Thunnus albacares*	.	.	.	.	.	.	.	.	.	G	.	.	.	A	.	.	.	.	.	.	.	.	.	.	.	.	T	.	.	T	.	.	.	.	.
DQ080288	*Thunnus albacares*	.	.	.	.	.	.	.	.	.	G	.	.	.	A	.	.	.	.	.	.	.	.	.	.	C	.	T	.	.	T	.	.	.	.	.
DQ497898	*Thunnus albacares*	.	.	.	.	.	.	.	.	.	G	.	.	.	A	.	.	.	.	.	.	.	.	.	.	.	.	T	.	.	T	.	.	.	C	.
DQ497899	*Thunnus albacares*	.	.	.	.	.	.	.	.	.	G	.	.	.	A	.	.	.	.	.	.	.	.	.	.	.	.	T	.	.	T	.	.	.	.	.
DQ497900	*Thunnus albacares*	.	.	.	.	.	.	.	.	.	G	.	.	.	A	.	.	.	.	.	.	.	.	.	.	.	.	T	.	.	T	.	.	.	C	.
DQ497901	*Thunnus albacares*	.	.	.	.	.	.	.	.	.	G	.	.	.	A	.	.	.	.	.	.	.	.	.	.	.	.	T	.	.	T	.	.	.	C	.
DQ497902	*Thunnus albacares*	.	.	.	.	.	.	.	.	.	G	.	.	.	A	.	.	.	.	.	.	.	.	.	.	.	.	T	.	.	T	.	.	.	C	.
DQ497903	*Thunnus albacares*	.	.	.	.	.	.	.	.	.	G	.	.	.	A	.	.	.	.	.	.	.	.	.	.	.	.	T	.	.	T	.	.	.	.	.
DQ497904	*Thunnus albacares*	.	.	.	.	.	.	.	.	.	G	.	.	.	A	.	.	.	.	.	.	.	.	.	.	.	.	T	.	.	T	.	.	.	.	.
EF141179	*Thunnus albacares*	.	.	.	.	.	.	.	.	.	G	.	.	.	A	.	.	.	.	.	.	.	.	.	.	.	.	T	.	.	T	.	.	.	C	.
EF392629	*Thunnus albacares*	.	.	.	.	.	.	.	.	.	G	.	.	T	A	.	.	.	.	.	.	.	.	.	.	.	.	T	.	.	T	.	.	.	.	.
EF392630	*Thunnus albacares*	.	.	.	.	.	.	.	.	.	G	.	.	.	A	.	.	.	.	.	.	.	.	.	.	.	.	T	.	.	T	.	.	.	.	.
EF456025	*Thunnus albacares*	.	.	.	.	.	.	.	.	.	G	.	.	.	A	.	.	.	.	.	.	.	.	.	.	.	.	T	.	.	T	.	.	.	.	.
EF456026	*Thunnus albacares*	.	.	.	.	.	.	.	.	.	G	.	.	.	A	.	.	.	.	.	.	.	.	.	.	.	.	T	.	.	T	.	.	.	C	.
EU250968	*Thunnus albacares*	.	.	.	.	.	.	.	.	.	G	.	.	.	A	.	.	.	.	.	.	.	.	.	.	C	.	T	.	.	T	.	.	.	.	.
EU250969	*Thunnus albacares*	.	.	.	.	.	.	.	.	.	G	.	.	.	A	.	.	.	.	.	.	.	.	.	.	.	.	T	.	.	T	.	.	.	C	.
EU250970	*Thunnus albacares*	.	.	.	.	.	.	.	.	.	G	.	.	.	A	.	.	.	.	.	.	.	.	.	.	.	.	T	.	.	T	.	.	.	C	.
EU250971	*Thunnus albacares*	.	.	.	.	.	.	.	.	.	G	.	.	.	A	.	.	.	.	.	.	.	.	.	.	.	.	T	.	.	T	.	.	.	.	.
EU250972	*Thunnus albacares*	.	.	.	.	.	.	.	.	.	G	.	.	.	A	.	.	.	.	.	.	.	.	.	.	.	.	T	.	.	T	.	.	.	C	.
EU250973	*Thunnus albacares*	.	.	.	.	.	.	.	.	.	G	.	.	.	A	.	.	.	.	.	.	.	.	.	.	.	.	T	.	.	T	.	.	.	C	.
EU250979	*Thunnus albacares*	.	.	.	.	.	.	.	.	.	G	.	.	.	A	.	.	.	.	.	.	.	.	.	.	.	.	T	.	.	T	.	.	.	C	.
EU250980	*Thunnus albacares*	.	.	.	.	.	.	.	.	.	G	.	.	.	A	.	.	.	.	.	.	.	.	.	.	.	.	T	.	.	T	.	.	.	.	.
EU250984	*Thunnus albacares*	.	.	.	.	.	.	.	.	.	G	.	.	.	A	.	.	.	.	.	.	.	.	.	.	.	.	T	.	.	T	.	.	.	C	.
EU250985	*Thunnus albacares*	.	.	.	.	.	.	.	.	.	G	.	.	.	A	.	.	.	.	.	.	.	.	.	.	.	.	T	.	.	T	.	.	.	.	.
EU250986	*Thunnus albacares*	.	.	.	.	.	.	.	.	.	G	.	.	.	A	.	.	.	.	.	.	.	.	.	.	.	.	T	.	.	T	.	.	.	C	.
EU427561	*Thunnus albacares*	.	.	.	.	.	.	.	.	.	G	.	.	.	A	.	.	.	.	.	.	.	.	.	.	.	.	T	.	.	T	.	.	.	.	.
KC522353	*Thunnus albacares*	.	.	.	.	.	.	.	.	.	G	.	.	.	A	.	.	.	.	.	.	.	.	.	.	.	.	T	.	.	T	.	.	.	.	.

Supplementary Material

		2	6	9	18	21	30	33	36	39	45	63	69	72	75	84	93	96	102	105	111	120	123	129	132	144	156	162	174	180	183	192	198	213	219	231
KU904413	*Thunnus albacares*	.	.	.	.	.	.	C	.	.	G	.	.	.	A	.	.	.	.	.	.	.	.	.	.	.	.	T	.	.	T	.	.	G	.	.
KX755257	*Thunnus albacares*	.	.	.	.	.	.	.	.	.	G	.	.	.	A	.	.	.	.	.	.	.	.	.	.	.	.	T	.	.	T	.	.	.	.	.
KX755258	*Thunnus albacares*	.	.	.	.	.	.	.	.	.	G	.	.	.	A	.	.	.	.	.	.	.	.	.	.	.	.	T	.	.	T	.	.	.	C	.
KX755259	*Thunnus albacares*	.	.	.	.	.	.	.	.	.	G	.	.	.	A	.	.	.	.	.	.	.	.	.	.	C	.	T	.	.	T	.	.	.	.	.
KX755260	*Thunnus albacares*	.	.	.	.	.	.	C	.	.	G	.	.	.	A	.	.	.	.	.	.	.	.	.	.	.	.	T	.	.	T	.	.	.	.	.
KX755261	*Thunnus albacares*	.	.	.	.	.	.	.	.	.	G	.	.	.	A	.	.	.	.	.	.	.	.	.	.	.	.	T	.	.	T	.	.	.	C	.
KX755262	*Thunnus albacares*	.	.	.	.	.	.	.	.	.	G	.	.	.	A	.	.	.	.	.	.	.	.	.	.	.	.	T	.	.	T	.	.	.	.	.
KX755263	*Thunnus albacares*	.	.	.	.	.	.	.	.	.	G	.	.	.	A	.	.	.	.	.	.	.	.	.	.	.	.	T	.	.	T	.	.	.	C	.
KX755264	*Thunnus albacares*	.	.	.	.	.	.	.	.	.	G	.	.	.	A	.	.	.	.	.	.	.	.	.	.	.	.	T	.	.	T	.	.	.	.	.
KX755265	*Thunnus albacares*	.	.	.	.	.	.	.	.	.	G	.	.	.	A	.	.	.	.	.	.	.	.	.	.	.	.	T	.	.	T	.	.	.	.	.
KX755266	*Thunnus albacares*	.	.	.	.	.	.	.	.	.	G	.	.	.	A	.	.	.	.	.	.	.	.	.	.	.	.	T	.	.	T	.	.	.	C	.
KX755267	*Thunnus albacares*	.	.	.	.	.	.	.	.	.	G	.	.	.	A	.	.	.	.	.	.	.	.	.	.	.	.	T	.	.	T	.	.	.	C	.
KX755268	*Thunnus albacares*	.	.	.	.	.	.	.	.	.	G	.	.	.	A	.	.	.	.	.	.	.	.	.	.	.	.	T	.	.	T	.	.	.	.	.
KX755269	*Thunnus albacares*	.	.	.	.	.	.	C	.	.	G	.	.	.	A	.	.	.	.	.	.	.	.	.	.	.	.	T	.	.	T	.	.	.	C	.
KX755270	*Thunnus albacares*	.	.	.	.	.	.	.	.	.	G	.	.	.	A	.	.	.	.	.	.	.	.	.	.	.	.	T	.	.	T	.	.	.	C	.
KX755271	*Thunnus albacares*	.	.	.	.	.	.	.	.	.	G	.	.	.	A	.	.	.	.	.	.	.	.	.	.	.	.	T	.	.	T	.	.	.	.	.
KX755272	*Thunnus albacares*	.	.	.	.	.	.	.	.	.	G	.	.	.	A	.	.	.	.	.	.	.	.	.	.	.	.	T	.	.	T	.	.	.	.	.
KX755273	*Thunnus albacares*	.	.	.	.	.	.	.	.	.	G	.	.	.	A	.	.	.	.	.	.	.	.	.	.	C	.	T	.	.	T	.	.	.	.	.
KX755274	*Thunnus albacares*	.	.	.	.	.	.	.	.	.	G	.	.	.	A	.	.	.	.	.	.	.	.	.	.	.	.	T	.	.	T	.	.	.	.	.
MG017682	*Thunnus albacares*	.	.	.	.	.	.	.	.	.	G	.	.	.	A	.	.	.	.	.	.	.	.	.	.	.	.	T	.	.	T	.	.	.	C	.
MG017683	*Thunnus albacares*	.	.	.	.	.	.	.	.	.	G	.	.	.	A	.	.	.	.	.	.	.	.	.	.	.	.	T	.	.	T	.	.	.	C	.
MG017684	*Thunnus albacares*	.	.	.	.	.	.	.	.	.	.	.	.	.	A	.	.	.	.	.	.	G	.	.	.	.	.	T	.	.	T	.	.	.	.	.
MG017685	*Thunnus albacares*	.	.	.	.	.	.	.	.	.	.	.	.	.	A	.	.	.	.	.	.	G	.	.	.	.	.	T	.	.	T	.	.	.	.	.
MG017686	*Thunnus albacares*	.	.	.	.	.	.	C	.	.	G	.	.	.	A	.	.	.	.	.	.	.	.	.	.	.	.	T	.	.	T	.	.	.	.	.
MG017687	*Thunnus albacares*	.	.	.	.	.	.	.	.	.	.	.	.	.	A	.	.	.	.	.	.	.	.	.	.	.	.	T	.	.	.	.	.	.	.	.
KM588080	*Thunnus albacares*	.	.	.	.	.	.	.	.	.	G	.	.	.	A	.	.	.	.	.	.	.	.	.	.	.	.	T	.	.	T	.	.	.	C	.
NC 014061	*Thunnus albacares*	.	.	.	.	.	.	.	.	.	G	.	.	.	A	.	.	.	.	.	.	.	.	.	.	.	.	T	.	.	T	.	.	.	.	.
GU256528	*Thunnus albacares*	.	.	.	.	.	.	.	.	.	G	.	.	.	A	.	.	.	.	.	.	.	.	.	.	.	.	T	.	.	T	.	.	.	.	.
JN086153	*Thunnus albacares*	.	.	.	.	.	.	.	.	.	G	.	.	.	A	.	.	.	.	.	.	.	.	.	.	.	.	T	.	.	T	.	.	.	.	.
KT724724	*Thunnus albacares*	.	.	.	.	.	.	C	.	.	G	.	.	.	A	.	.	.	.	.	.	.	.	.	.	.	.	T	.	.	T	.	.	.	.	.
	YFT-AO-L1-1	.	.	.	.	.	.	.	.	.	G	.	.	.	A	.	.	.	.	.	.	.	.	.	.	.	.	T	.	.	T	.	.	.	C	C
	YFT-AO-L1-2	.	.	.	.	.	.	.	.	.	G	.	.	.	A	.	.	.	.	.	.	.	.	.	.	.	.	T	.	.	T	.	.	.	C	C
	YFT-AO-L1-3	.	.	.	.	.	.	.	.	.	G	.	.	.	A	.	.	.	.	.	.	.	.	.	.	.	.	T	.	.	T	.	.	.	C	C
	YFT-IO-L1-1	.	.	.	.	.	.	.	.	.	G	.	.	.	A	.	.	.	.	.	.	.	.	.	.	.	.	T	.	.	T	.	.	.	C	C
	YFT-IO-L1-2	.	.	.	.	.	.	.	.	.	G	.	.	.	A	.	.	.	.	.	.	.	.	.	.	.	.	T	.	.	T	.	.	.	C	C
	YFT-IO-L1-3	.	.	.	.	.	.	.	.	.	G	.	.	.	A	.	.	.	.	.	.	.	.	.	.	.	.	T	.	.	T	.	.	.	C	C
	YFT-WCPO-L1-1	.	.	.	.	.	.	.	.	.	G	.	.	.	A	.	.	.	.	.	.	.	.	.	.	.	.	T	.	.	T	.	.	.	C	C
	YFT-WCPO-L1-2	.	.	.	.	.	.	.	.	.	G	.	.	.	A	.	.	.	.	.	.	.	.	.	.	.	.	T	.	.	T	.	.	.	C	C
	YFT-WCPO-L1-3	.	.	.	.	.	.	.	.	.	G	.	.	.	A	.	.	.	.	.	.	.	.	.	.	.	.	T	.	.	T	.	.	.	C	C
	YFT-EPO-L1-1	.	.	.	.	.	.	.	.	.	G	.	.	.	A	.	.	.	.	.	.	.	.	.	.	C	.	T	.	.	T	.	.	.	C	C
	YFT-EPO-L1-2	.	.	.	.	.	.	.	.	.	G	.	.	.	A	.	.	.	.	.	.	.	.	.	.	C	.	T	.	.	T	.	.	.	C	C
	YFT-EPO-L1-3	.	.	.	.	.	.	.	.	.	G	.	.	.	A	.	.	.	.	.	.	.	.	.	.	C	.	T	.	.	T	.	.	.	C	C

Supplementary Material

		2	6	9	18	21	30	33	36	39	45	63	69	72	75	84	93	96	102	105	111	120	123	129	132	144	156	162	174	180	183	192	198	213	219	231
DQ080272	*Thunnus obesus*	.	.	.	.	.	.	.	.	.	.	.	.	A	A	.	.	.	C	.	.	.	.	.	.	.	.	T	.	.	.	C	.	.	.	.
DQ080273	*Thunnus obesus*	.	.	.	.	.	.	.	.	.	.	.	.	A	A	.	.	.	C	.	.	.	.	.	.	.	.	T	.	C	.	C	.	.	.	.
DQ080274	*Thunnus obesus*	.	.	.	.	.	.	.	.	.	.	.	.	A	A	.	.	.	C	.	.	.	.	.	.	.	.	T	.	.	.	C	.	.	.	.
DQ080275	*Thunnus obesus*	.	.	.	.	.	.	.	.	.	.	.	.	A	A	.	.	.	C	.	.	.	.	.	.	.	.	T	.	.	.	C	.	.	.	.
DQ080276	*Thunnus obesus*	.	.	.	.	.	.	.	.	.	.	.	.	A	A	.	.	.	.	.	.	.	.	.	.	.	.	T	.	.	.	.	.	G	.	.
DQ080277	*Thunnus obesus*	.	.	.	.	.	.	.	.	.	.	.	.	A	A	.	.	.	.	.	.	.	.	.	.	.	.	T	.	.	.	.	.	G	.	.
DQ080278	*Thunnus obesus*	.	.	.	.	.	.	.	.	.	.	.	.	A	A	.	.	.	.	.	.	.	.	.	.	.	.	T	.	.	.	.	.	G	.	.
DQ080279	*Thunnus obesus*	.	.	.	.	.	.	.	.	.	.	.	.	A	A	.	.	.	C	.	.	.	.	.	.	.	.	T	.	.	.	C	.	.	.	.
DQ080280	*Thunnus obesus*	.	.	.	.	.	.	.	.	.	.	.	.	A	A	.	.	.	C	.	.	.	.	.	.	.	.	T	.	.	.	C	.	.	.	.
DQ198013	*Thunnus obesus*	.	.	.	.	.	.	.	.	.	.	.	.	A	A	.	.	.	C	.	.	.	.	.	.	.	.	T	.	.	.	C	.	.	.	.
DQ497910	*Thunnus obesus*	.	.	.	.	.	.	.	.	.	.	.	.	A	A	.	.	.	C	.	.	.	.	.	.	.	.	T	.	.	.	C	.	.	.	.
DQ497911	*Thunnus obesus*	.	.	.	.	.	.	.	.	.	.	.	.	A	A	.	.	.	C	.	.	.	.	.	.	.	C	T	.	.	.	C	.	.	.	.
DQ497912	*Thunnus obesus*	.	.	.	.	.	.	.	.	.	.	.	.	A	A	.	.	.	C	.	.	.	.	.	.	.	.	T	.	.	.	C	.	.	.	.
EF141180	*Thunnus obesus*	.	.	.	.	.	.	.	.	.	G	.	.	.	A	.	.	.	.	.	.	.	.	.	.	.	.	T	.	.	T	.	.	.	.	.
EF392631	*Thunnus obesus*	.	.	.	.	.	.	.	.	.	.	.	.	A	A	.	.	.	C	.	.	.	.	.	.	.	.	T	.	.	.	C	.	.	.	.
EF392632	*Thunnus obesus*	.	.	.	.	.	.	.	.	.	.	.	.	A	A	.	.	.	.	.	.	.	.	.	.	.	.	T	.	.	.	.	.	G	.	.
EF439240	*Thunnus obesus*	.	.	.	.	.	.	.	.	.	.	.	.	A	A	.	.	.	.	.	.	.	.	.	.	.	.	T	.	.	.	.	.	G	.	.
EF439241	*Thunnus obesus*	.	.	.	.	.	.	.	.	.	.	.	.	A	A	.	.	.	.	.	.	.	.	.	.	.	.	T	.	.	.	.	.	G	.	.
EF456027	*Thunnus obesus*	.	.	.	.	.	.	.	.	.	G	.	.	.	A	.	.	.	.	.	.	.	.	.	.	.	.	T	.	.	T	.	.	.	.	.
EF456028	*Thunnus obesus*	.	.	.	.	.	.	.	.	.	G	.	.	.	A	.	.	.	.	.	.	.	.	.	.	.	.	T	.	.	T	.	.	.	.	.
EU224036	*Thunnus obesus*	.	.	.	.	.	.	.	.	.	.	.	.	A	A	.	.	.	.	.	.	.	.	.	.	.	.	T	.	.	.	.	.	G	.	.
EU349400	*Thunnus obesus*	.	.	.	.	.	.	.	.	.	.	.	.	A	A	.	.	.	C	.	.	.	.	.	.	.	.	T	.	.	.	C	.	.	.	.
EU349401	*Thunnus obesus*	.	.	.	.	.	.	.	.	.	.	.	.	A	A	.	.	.	C	.	.	.	.	.	.	.	.	T	.	.	.	C	.	.	.	.
EU349402	*Thunnus obesus*	.	.	.	.	.	.	.	.	.	.	.	.	A	A	.	.	.	C	.	.	.	.	.	.	.	.	T	.	.	.	C	.	.	.	.
EU935767	*Thunnus obesus*	.	.	.	.	.	.	.	.	.	.	.	.	A	A	.	.	.	C	.	.	.	.	.	.	.	.	T	.	.	.	C	.	.	.	.
EU935768	*Thunnus obesus*	.	.	.	.	.	.	.	.	.	.	.	.	A	A	.	.	.	C	.	.	.	.	.	.	.	.	T	.	.	.	C	.	.	.	.
EU935769	*Thunnus obesus*	.	.	.	.	.	.	.	.	.	.	.	.	A	A	.	.	.	C	.	.	.	.	.	.	.	.	T	.	.	.	C	.	.	.	.
EU935770	*Thunnus obesus*	.	.	.	.	.	.	.	.	.	.	.	.	A	A	.	.	.	C	.	.	.	.	.	.	.	.	T	.	.	.	C	.	.	.	.
EU935771	*Thunnus obesus*	.	.	.	.	.	.	.	.	.	.	.	.	A	A	.	.	.	C	.	.	.	.	.	.	.	.	T	.	.	.	C	.	.	.	.
EU935772	*Thunnus obesus*	.	.	.	.	.	.	.	.	.	.	.	.	A	A	.	.	.	C	.	.	.	.	.	.	.	.	T	.	.	.	C	.	.	.	.
EU935773	*Thunnus obesus*	.	.	.	.	.	.	.	.	.	.	.	.	A	A	.	.	.	C	.	.	.	.	.	.	.	.	T	.	.	.	C	.	.	.	.
GU256525	*Thunnus obesus*	.	.	.	.	.	.	.	.	.	.	.	.	A	A	.	.	.	.	.	.	.	.	.	.	.	.	T	.	.	.	.	.	G	.	.
KY400011	*Thunnus obesus*	.	.	.	.	.	.	.	.	.	.	.	.	A	A	.	.	.	.	.	.	.	.	.	.	.	.	T	.	.	.	.	.	G	.	.
NC 014059	*Thunnus obesus*	.	.	.	.	.	.	.	.	.	.	.	.	A	A	.	.	.	.	.	.	.	.	.	.	.	.	T	.	.	.	.	.	G	.	.
JN086152	*Thunnus obesus*	.	.	.	.	.	.	.	.	.	.	.	.	A	A	.	.	.	C	.	.	.	.	.	.	.	.	T	.	C	.	C	.	.	.	.
KC522356	*Thunnus obesus*	.	.	.	.	.	.	.	.	.	.	.	.	A	A	.	.	.	C	.	.	.	.	.	.	.	.	T	.	.	.	C	.	.	.	.
MG017688	*Thunnus obesus*	.	.	.	.	.	.	.	.	.	.	.	.	A	A	.	.	.	C	.	.	.	.	.	.	.	.	T	.	.	.	C	.	.	.	.
MG017689	*Thunnus obesus*	.	.	.	.	.	.	.	.	.	.	.	.	A	A	.	.	.	C	.	.	.	.	.	.	.	.	T	.	.	.	C	.	.	.	.
MG017690	*Thunnus obesus*	.	.	.	.	.	.	.	.	.	.	.	.	A	A	.	.	.	C	.	.	.	.	.	.	.	.	T	.	.	.	C	.	.	.	.
MG017691	*Thunnus obesus*	.	.	.	.	.	.	.	.	.	.	.	.	A	A	.	.	.	C	.	.	.	.	.	.	.	.	T	.	.	.	C	.	.	.	.
MG017692	*Thunnus obesus*	.	.	.	.	.	.	.	.	.	.	.	.	A	A	.	.	.	C	.	.	.	.	.	.	.	.	T	.	.	.	C	.	.	.	.
MG017693	*Thunnus obesus*	.	.	.	.	.	.	.	.	.	.	.	.	A	A	.	.	.	C	.	.	.	.	.	.	.	.	T	.	C	.	C	.	.	.	.

Supplementary Material

		2	6	9	18	21	30	33	36	39	45	63	69	72	75	84	93	96	102	105	111	120	123	129	132	144	156	162	174	180	183	192	198	213	219	231
MG017694	*Thunnus obesus*	.	.	.	.	.	.	.	.	.	.	.	.	A	A	.	.	.	C	.	.	.	.	.	.	.	.	T	.	.	.	C	.	.	.	.
MG017695	*Thunnus obesus*	.	.	.	.	.	.	.	.	.	.	.	.	A	A	.	.	.	C	.	.	.	.	.	.	.	.	T	.	.	.	C	.	.	.	.
MG017696	*Thunnus obesus*	.	.	.	.	.	.	.	.	.	.	.	.	A	A	.	.	.	C	.	.	.	.	.	.	.	.	T	.	.	.	C	.	.	.	.
MG017697	*Thunnus obesus*	.	.	.	.	.	.	.	.	.	.	.	.	A	A	.	.	.	C	.	.	.	.	.	.	.	.	T	.	C	.	C	.	.	.	.
	BET-IO-L1-1	.	.	.	.	.	.	.	.	.	.	.	.	A	A	.	.	.	C	.	.	.	.	.	.	.	.	T	.	.	.	C	.	.	C	C
	BET-IO-L1-2	.	.	.	.	.	.	.	.	.	.	.	.	A	A	.	.	.	C	.	.	.	.	.	.	.	.	T	.	.	.	C	.	.	C	C
	BET-IO-L1-3	.	.	.	.	.	.	.	.	.	.	.	.	A	A	.	.	.	C	.	.	.	.	.	.	.	.	T	.	.	.	C	.	.	C	C
	BET-EPO-L1-1	.	.	.	.	.	.	.	.	.	.	.	.	A	A	.	.	.	C	.	.	.	.	.	.	.	.	T	.	.	.	C	.	.	C	C
	BET-EPO-L1-2	.	.	.	.	.	.	.	.	.	.	.	.	A	A	.	.	.	C	.	.	.	.	.	.	.	.	T	.	.	.	C	.	.	C	C
	BET-EPO-L1-3	.	.	.	.	.	.	.	.	.	.	.	.	A	A	.	.	.	C	.	.	.	.	.	.	.	.	T	.	.	.	C	.	.	C	C
DQ197958	*Katsuwonus pelamis*	C	T	T	T	C	T	C	.	C	.	T	C	A	T	A	T	T	C	.	A	.	G	.	A	.	C	T	A	C	A	C	C	.	.	C
EF392592	*Katsuwonus pelamis*	C	T	T	T	C	T	C	.	C	.	T	C	A	T	A	T	T	C	.	A	.	G	.	A	.	C	T	A	C	A	C	C	.	.	C
EF392591	*Katsuwonus pelamis*	C	T	T	T	C	T	C	.	C	.	.	C	A	T	A	T	T	C	.	A	.	G	.	A	.	C	T	A	C	A	C	C	.	.	C
EF439209	*Katsuwonus pelamis*	C	T	T	T	C	T	C	.	C	.	T	C	A	T	A	T	T	C	.	A	.	.	.	A	.	C	T	A	C	A	C	C	.	.	C
EF439208	*Katsuwonus pelamis*	C	T	T	T	C	T	C	.	C	.	T	C	A	T	A	T	T	C	.	A	.	.	.	A	.	C	T	A	C	A	C	C	.	.	C
EF427567	*Katsuwonus pelamis*	C	T	T	T	C	T	C	.	C	.	T	C	A	T	A	T	T	C	.	A	.	G	.	A	.	C	T	A	C	A	C	C	.	.	C
EF427566	*Katsuwonus pelamis*	C	T	T	T	C	T	C	.	C	.	T	C	A	T	A	T	T	C	.	A	.	G	.	A	.	C	T	A	C	A	C	C	.	.	C
KP669172	*Katsuwonus pelamis*	C	T	T	T	C	T	C	.	C	.	T	C	A	T	A	T	T	C	.	A	.	G	.	A	.	C	T	A	C	A	C	C	.	.	C
KP669171	*Katsuwonus pelamis*	C	T	T	T	C	T	.	.	C	.	T	C	A	T	A	T	T	C	T	A	.	G	.	A	.	C	T	A	C	A	C	C	.	.	C
KP669170	*Katsuwonus pelamis*	C	T	T	T	C	T	C	.	C	.	T	C	A	T	A	T	T	C	T	A	.	.	.	A	.	C	T	A	C	A	C	C	G	.	C
KP669169	*Katsuwonus pelamis*	C	.	T	T	C	T	C	.	C	.	T	C	A	T	A	T	T	C	.	A	.	.	.	A	.	C	T	A	C	A	C	C	G	.	C
KP669168	*Katsuwonus pelamis*	C	T	T	T	C	T	C	.	C	.	T	C	A	T	A	T	T	C	.	A	.	G	.	A	.	C	T	A	C	A	C	C	.	.	C
KP669167	*Katsuwonus pelamis*	C	T	T	T	C	T	C	.	C	.	T	C	A	T	A	T	T	C	.	A	.	G	.	A	.	C	T	A	C	A	C	C	.	.	C
KP669166	*Katsuwonus pelamis*	C	T	T	T	C	T	C	.	C	.	T	C	A	T	A	T	T	C	.	A	.	G	.	A	.	C	T	A	C	A	C	C	.	.	C
KP669165	*Katsuwonus pelamis*	C	T	T	T	C	T	C	.	C	.	T	C	A	T	A	T	T	C	.	A	.	.	.	A	.	C	T	A	C	A	C	C	G	.	C
KP669164	*Katsuwonus pelamis*	C	T	T	T	C	T	C	.	C	.	T	C	A	T	A	T	T	C	.	A	.	G	A	A	.	C	T	A	C	A	C	C	.	.	C
KP669163	*Katsuwonus pelamis*	C	T	T	T	C	.	C	.	C	.	T	C	A	T	A	T	T	C	.	A	.	G	.	A	.	C	T	A	C	A	C	C	G	.	C
KP669162	*Katsuwonus pelamis*	C	T	T	T	C	T	C	.	C	.	T	C	A	T	A	T	T	C	T	A	.	.	.	A	.	C	T	A	C	A	C	C	G	.	C
KP669161	*Katsuwonus pelamis*	C	T	T	T	C	T	C	.	C	.	T	C	A	T	A	T	T	C	.	A	.	G	.	A	.	C	T	A	C	A	C	C	.	.	C
KP669160	*Katsuwonus pelamis*	C	T	T	T	C	T	C	.	C	.	T	C	A	T	A	T	T	C	.	A	.	G	.	A	.	C	T	A	C	A	C	C	.	.	C
KP669159	*Katsuwonus pelamis*	C	T	T	T	C	T	C	.	C	.	T	C	A	T	A	T	T	C	T	A	.	.	.	A	.	C	T	A	C	A	C	C	.	.	C
KP669158	*Katsuwonus pelamis*	C	T	T	T	C	T	C	.	C	.	T	C	A	T	A	T	T	C	T	A	.	.	.	A	.	C	T	A	C	A	C	C	G	.	C
KP669157	*Katsuwonus pelamis*	C	T	T	T	C	T	C	.	C	.	T	C	A	C	A	T	T	C	T	A	.	.	A	A	.	C	T	A	C	A	C	C	.	.	C
KP669156	*Katsuwonus pelamis*	C	T	T	T	C	T	C	.	C	.	T	C	A	T	A	T	T	C	.	A	.	G	.	A	.	C	T	A	C	A	C	C	.	.	C
KP669155	*Katsuwonus pelamis*	C	T	T	T	C	T	C	.	C	.	T	C	A	T	A	T	T	C	T	A	.	.	.	A	.	C	T	A	C	A	C	C	G	.	.
KP669154	*Katsuwonus pelamis*	C	T	T	T	C	T	C	.	C	.	T	C	A	T	A	T	T	C	T	A	.	.	.	A	.	C	T	A	C	A	C	C	.	.	C
KP669153	*Katsuwonus pelamis*	C	T	T	T	C	T	C	.	C	.	T	C	A	T	A	T	T	C	T	A	.	.	.	A	.	C	T	A	C	A	C	C	.	.	.
KP669152	*Katsuwonus pelamis*	C	T	T	T	C	T	C	.	C	.	T	C	A	T	A	T	T	C	.	A	.	G	.	A	.	C	T	A	C	A	C	C	G	.	C
KP669151	*Katsuwonus pelamis*	C	T	T	T	C	T	C	.	C	.	T	C	A	T	A	T	T	C	.	A	.	G	.	A	.	C	T	A	C	A	C	C	.	.	C
KP669150	*Katsuwonus pelamis*	C	T	T	T	C	T	C	.	C	.	T	C	A	T	A	T	T	C	T	A	.	.	.	A	.	C	T	A	C	A	C	C	G	.	C
KP669149	*Katsuwonus pelamis*	C	T	T	T	C	T	C	.	C	.	T	C	A	T	A	T	T	C	T	A	.	.	.	A	.	C	T	A	C	A	C	C	G	.	C
KP669148	*Katsuwonus pelamis*	C	T	T	T	C	T	C	.	C	.	T	C	A	T	A	T	T	C	.	G	.	G	.	A	.	C	T	A	C	A	C	C	.	.	C

Supplementary Material

		2	6	9	18	21	30	33	36	39	45	63	69	72	75	84	93	96	102	105	111	120	123	129	132	144	156	162	174	180	183	192	198	213	219	231
KP669147	*Katsuwonus pelamis*	C	T	T	T	C	T	C	.	C	.	T	C	A	T	A	T	T	C	T	A	.	.	.	A	.	C	T	A	C	A	C	C	G	.	C
KP669146	*Katsuwonus pelamis*	C	T	T	T	C	T	C	.	C	.	T	C	A	T	A	T	T	C	T	A	.	.	.	A	.	C	T	A	C	A	C	C	G	.	C
KP669145	*Katsuwonus pelamis*	C	T	T	T	C	T	C	.	C	.	T	C	A	T	A	T	T	C	.	A	.	G	.	A	.	C	T	A	C	A	C	C	G	.	C
KP669144	*Katsuwonus pelamis*	C	T	T	T	C	T	C	.	C	.	T	C	A	T	A	T	T	C	.	A	.	G	.	A	.	C	T	A	C	A	C	C	.	.	C
KP669143	*Katsuwonus pelamis*	C	T	T	T	C	T	A	.	C	.	T	C	A	T	A	T	T	C	.	A	.	.	.	A	.	C	T	A	C	A	C	C	G	.	C
KP669142	*Katsuwonus pelamis*	C	T	T	T	C	T	C	.	C	.	T	C	A	T	A	T	T	C	.	A	.	G	.	A	.	C	T	A	C	A	C	C	.	.	C
KP669141	*Katsuwonus pelamis*	C	T	T	T	C	T	C	.	C	.	T	C	A	T	A	T	T	C	T	A	.	.	.	A	.	C	T	A	C	A	C	C	G	.	C
KP669140	*Katsuwonus pelamis*	C	T	T	T	C	T	C	.	C	.	T	C	A	T	A	T	T	C	T	A	.	.	.	A	.	C	T	A	C	A	C	C	.	.	C
KP669139	*Katsuwonus pelamis*	C	T	T	T	C	T	C	.	C	.	T	C	A	T	A	T	T	C	.	A	.	G	.	A	.	C	T	A	C	A	C	C	.	.	C
KP669138	*Katsuwonus pelamis*	C	T	T	T	C	T	C	.	C	.	T	T	A	T	A	T	T	C	.	A	.	G	.	A	.	C	T	A	C	A	C	C	.	.	C
KP669137	*Katsuwonus pelamis*	C	T	T	T	C	T	C	.	C	.	T	C	A	T	A	T	T	C	T	A	.	G	.	A	.	C	T	A	C	A	C	C	.	.	C
KP669136	*Katsuwonus pelamis*	C	T	T	T	C	T	C	.	C	.	C	C	A	T	A	T	T	C	.	A	.	.	A	A	.	C	T	A	C	A	C	C	G	.	C
KP669135	*Katsuwonus pelamis*	C	T	T	.	C	T	C	.	C	.	T	C	A	T	A	T	T	C	T	A	.	.	.	A	.	C	T	A	C	A	C	C	G	.	C
KP669134	*Katsuwonus pelamis*	C	T	T	T	C	T	C	.	C	.	T	C	A	T	A	T	T	C	.	A	.	G	.	A	.	C	T	A	C	A	C	C	G	.	C
KP669133	*Katsuwonus pelamis*	C	T	T	T	C	T	C	.	C	.	T	C	A	T	A	T	T	C	.	A	.	G	.	A	.	C	T	A	C	A	C	C	G	.	C
KP669132	*Katsuwonus pelamis*	C	T	T	T	C	T	C	.	C	.	T	C	A	T	A	T	T	C	.	A	.	G	.	A	.	C	T	A	C	A	C	C	.	C	C
KP669131	*Katsuwonus pelamis*	C	T	T	T	C	T	C	.	C	.	T	C	A	T	A	T	T	C	T	A	.	.	.	A	.	C	T	A	C	A	C	C	G	.	C
KP669130	*Katsuwonus pelamis*	C	T	T	T	C	T	C	.	C	.	T	C	A	T	A	T	T	C	.	A	.	.	.	A	.	C	T	A	C	A	C	C	.	.	C
KP669129	*Katsuwonus pelamis*	C	T	T	T	C	T	C	.	C	.	T	C	A	T	A	T	T	C	.	A	.	G	.	A	.	C	T	A	C	A	C	C	G	.	C
KP669128	*Katsuwonus pelamis*	C	T	T	T	C	T	C	.	C	.	T	C	A	T	A	T	T	C	.	A	.	.	.	A	.	C	T	A	C	A	C	C	.	.	C
KP669127	*Katsuwonus pelamis*	C	T	T	T	C	T	C	.	C	.	T	.	A	T	A	T	T	C	T	A	.	.	.	A	.	C	T	A	C	A	C	C	G	.	C
KP669126	*Katsuwonus pelamis*	C	T	T	T	C	T	C	.	C	.	T	C	A	T	A	T	T	C	T	A	.	.	.	A	.	C	T	A	C	A	C	C	.	.	C
KP669125	*Katsuwonus pelamis*	C	T	T	T	C	T	C	.	C	.	T	C	A	T	A	T	.	C	T	A	.	.	.	A	.	C	T	A	C	A	C	C	G	.	C
KP669124	*Katsuwonus pelamis*	C	T	T	T	C	T	C	.	C	.	T	C	A	T	A	T	T	C	T	A	.	.	.	A	.	C	T	A	C	A	C	C	G	.	C
KP669123	*Katsuwonus pelamis*	C	T	T	T	C	T	C	.	C	.	T	C	A	T	A	T	T	C	T	A	.	.	.	A	.	C	T	A	C	A	C	C	.	.	C
KP669122	*Katsuwonus pelamis*	C	T	T	T	C	T	C	.	C	.	T	C	A	T	A	T	T	C	T	A	.	.	.	A	.	C	T	A	C	A	C	C	G	.	C
KP669121	*Katsuwonus pelamis*	C	.	T	T	C	T	C	.	C	.	T	C	A	T	A	T	T	C	.	A	.	.	.	A	.	C	T	A	C	A	C	C	G	.	C
KP669120	*Katsuwonus pelamis*	C	T	T	T	C	T	C	.	C	.	T	C	A	T	A	T	T	C	.	A	.	G	.	A	.	C	T	A	C	A	C	C	G	.	C
KP669119	*Katsuwonus pelamis*	C	T	T	T	C	T	C	.	C	.	T	C	A	T	A	T	T	C	.	A	.	G	.	A	.	C	T	A	C	A	C	C	.	.	C
KP669118	*Katsuwonus pelamis*	C	T	T	T	C	T	C	.	C	.	T	C	A	T	A	T	T	C	.	A	.	.	.	A	.	C	T	A	C	A	C	C	G	.	C
KP669117	*Katsuwonus pelamis*	C	T	T	T	C	T	C	.	C	.	C	C	A	T	A	T	T	C	.	A	.	.	.	A	.	C	T	A	C	A	C	C	G	.	C
KP669116	*Katsuwonus pelamis*	C	.	T	T	C	T	C	.	C	.	T	C	A	T	A	T	T	C	.	A	.	.	.	A	.	C	T	A	C	A	C	C	.	.	C
KP669115	*Katsuwonus pelamis*	C	T	T	T	C	T	C	.	C	.	T	C	A	T	A	T	T	C	T	A	.	.	A	A	.	C	T	A	C	A	C	C	.	.	.
KP669114	*Katsuwonus pelamis*	C	T	T	C	C	T	C	.	C	.	T	C	A	T	A	T	T	C	T	A	.	.	.	A	.	C	T	A	C	A	C	C	G	.	C
KP669113	*Katsuwonus pelamis*	C	T	T	T	C	T	C	.	C	.	T	C	A	T	A	T	T	C	T	A	.	.	.	A	.	C	T	A	C	A	C	C	.	.	C
KP669112	*Katsuwonus pelamis*	C	T	T	T	C	T	C	.	C	.	T	C	A	T	A	T	T	C	.	A	.	G	.	A	.	C	T	A	C	A	C	C	.	.	C
KP669111	*Katsuwonus pelamis*	C	T	T	T	C	T	C	.	C	.	T	C	A	T	A	T	T	C	T	A	.	.	.	A	.	C	T	A	C	A	C	C	G	.	C
KP669110	*Katsuwonus pelamis*	C	T	T	T	C	T	C	.	C	.	T	C	A	T	A	T	T	C	.	A	.	G	.	A	.	C	T	A	C	A	C	C	G	.	C
KP669109	*Katsuwonus pelamis*	C	T	T	T	C	T	C	.	C	.	T	C	A	T	A	T	T	C	.	A	.	G	.	A	.	C	T	A	C	A	C	C	.	.	C
KP669108	*Katsuwonus pelamis*	C	T	T	T	C	T	C	.	C	.	T	C	A	T	A	T	T	C	.	A	.	G	.	A	.	C	T	A	C	A	C	C	G	.	C
KP669107	*Katsuwonus pelamis*	C	T	T	T	C	T	C	.	C	.	T	C	A	T	A	T	T	C	.	A	.	G	.	A	.	C	T	A	C	A	C	C	G	.	C
KP669106	*Katsuwonus pelamis*	C	T	T	T	C	T	C	.	C	.	T	C	A	T	A	T	T	C	.	A	.	G	.	A	.	C	T	A	C	A	C	C	.	.	C

Supplementary Material

		2	6	9	18	21	30	33	36	39	45	63	69	72	75	84	93	96	102	105	111	120	123	129	132	144	156	162	174	180	183	192	198	213	219	231
KP669105	*Katsuwonus pelamis*	C	T	T	T	C	T	C	.	C	.	T	C	A	T	A	T	T	C	.	A	.	G	.	A	.	C	T	A	C	A	C	C	.	.	C
KP669104	*Katsuwonus pelamis*	C	T	T	T	C	.	C	.	C	.	T	C	A	T	A	T	T	C	T	A	.	.	.	A	.	C	T	A	C	A	C	C	.	.	C
KP669103	*Katsuwonus pelamis*	C	T	T	T	C	T	C	.	C	.	T	C	A	T	A	T	T	C	.	A	.	.	.	A	.	C	T	A	C	A	C	C	.	.	C
KP669102	*Katsuwonus pelamis*	C	T	T	T	C	T	C	.	C	.	T	C	A	T	A	T	T	C	.	A	.	G	.	A	.	C	T	A	C	A	C	C	.	.	C
KP669101	*Katsuwonus pelamis*	C	T	T	T	.	T	C	.	C	.	T	C	A	T	A	T	T	C	T	A	.	.	.	A	.	C	T	A	C	A	C	C	G	.	C
KP669100	*Katsuwonus pelamis*	C	T	T	T	C	T	C	.	C	.	T	C	A	T	A	T	T	C	.	A	.	.	.	A	.	C	T	A	C	A	C	C	.	.	C
KJ617389	*Katsuwonus pelamis*	C	T	T	T	C	T	C	.	C	.	T	C	A	T	A	T	T	C	T	A	.	.	.	A	.	A	T	A	C	A	C	C	G	.	C
KJ617388	*Katsuwonus pelamis*	C	T	T	C	C	T	C	.	C	.	T	C	A	T	A	T	T	C	T	A	.	.	.	A	.	C	T	A	C	A	C	C	G	.	C
KJ617387	*Katsuwonus pelamis*	C	T	T	T	C	T	C	.	C	.	T	C	A	T	A	T	T	C	T	A	.	.	.	A	.	C	T	A	C	A	C	C	G	.	C
KJ617386	*Katsuwonus pelamis*	C	T	T	T	C	T	C	.	C	.	T	C	A	T	A	T	T	C	T	A	.	.	.	A	.	C	T	A	C	A	C	C	.	.	C
KJ617385	*Katsuwonus pelamis*	C	.	T	T	C	T	C	.	C	.	T	C	A	T	A	T	T	C	.	A	.	.	.	A	.	C	T	A	C	A	C	C	G	.	C
KJ617384	*Katsuwonus pelamis*	C	.	T	T	C	T	C	.	C	.	T	C	A	T	A	T	T	C	.	A	.	.	.	A	.	C	T	A	C	A	C	C	G	.	C
KJ617383	*Katsuwonus pelamis*	C	.	T	T	C	T	C	.	C	.	T	C	A	T	A	T	T	C	.	A	.	.	.	A	.	C	T	A	C	A	C	C	G	.	C
KJ617382	*Katsuwonus pelamis*	C	.	T	T	C	T	C	.	C	.	T	C	A	T	A	T	T	C	.	A	.	.	.	A	.	C	T	A	C	A	C	C	G	.	C
KJ617381	*Katsuwonus pelamis*	C	.	T	T	C	.	C	.	C	.	T	C	A	T	A	T	T	C	.	A	.	.	.	A	.	C	T	A	C	A	C	C	G	.	C
KJ617380	*Katsuwonus pelamis*	C	.	T	T	C	T	C	.	C	.	T	C	A	T	A	T	T	C	.	A	.	.	.	A	.	C	T	A	C	A	C	C	G	.	C
KJ617379	*Katsuwonus pelamis*	C	.	T	T	C	T	C	.	C	.	T	C	A	T	A	T	T	C	.	A	.	.	.	A	.	C	T	A	C	A	C	.	G	.	C
KJ617378	*Katsuwonus pelamis*	C	.	T	T	C	T	C	.	C	.	T	C	A	T	A	T	T	C	.	A	.	.	.	A	.	C	T	A	C	A	C	C	G	.	C
KJ617377	*Katsuwonus pelamis*	C	.	T	T	C	T	C	.	C	.	T	C	A	T	A	T	T	C	.	A	.	.	.	A	.	C	T	A	C	A	C	C	G	.	C
KJ617376	*Katsuwonus pelamis*	C	T	T	T	C	T	C	.	C	.	T	C	A	T	A	T	T	C	.	A	.	G	.	A	.	C	T	A	C	A	C	C	.	.	C
KJ617375	*Katsuwonus pelamis*	C	T	T	T	C	T	C	.	C	.	T	C	A	T	A	T	T	C	.	A	.	.	.	A	.	C	T	A	C	A	C	C	G	.	C
KJ617374	*Katsuwonus pelamis*	C	T	T	T	C	T	C	.	C	.	T	C	A	T	A	T	T	C	.	A	.	.	.	A	.	C	T	A	C	A	C	C	G	.	C
KJ617373	*Katsuwonus pelamis*	C	T	T	T	C	T	C	.	C	.	T	C	A	T	A	T	T	C	.	A	.	G	.	A	.	C	T	A	C	A	C	C	.	.	C
KJ617372	*Katsuwonus pelamis*	C	T	T	T	C	T	C	.	C	.	T	C	A	T	A	T	T	C	.	A	.	G	.	A	.	C	T	A	C	A	C	C	G	.	C
KJ617371	*Katsuwonus pelamis*	C	T	T	T	C	T	C	.	C	.	T	C	A	T	A	T	T	C	.	A	.	.	.	A	.	C	T	A	C	A	C	C	.	.	C
KJ617370	*Katsuwonus pelamis*	C	T	T	T	C	T	C	.	C	.	T	C	A	T	A	T	T	C	.	A	.	.	.	A	.	C	T	A	C	A	C	C	.	.	C
KJ617369	*Katsuwonus pelamis*	C	T	T	T	C	T	C	.	C	.	T	C	A	T	A	T	T	C	.	A	.	G	.	A	.	C	T	A	C	A	C	.	.	.	C
KJ617368	*Katsuwonus pelamis*	C	T	T	T	C	T	C	.	C	.	T	C	A	T	A	T	T	C	.	A	.	G	.	A	.	C	T	A	C	A	C	C	.	.	C
KJ617367	*Katsuwonus pelamis*	C	T	T	T	C	T	C	.	C	.	T	C	A	T	A	T	T	C	.	A	.	G	.	A	.	C	T	A	C	A	C	C	.	.	C
KJ617366	*Katsuwonus pelamis*	C	T	T	T	C	T	C	.	C	.	T	C	A	C	A	T	T	C	.	A	.	G	.	A	.	C	T	A	C	A	C	C	.	.	C
KJ617365	*Katsuwonus pelamis*	C	T	T	T	C	T	C	.	C	.	T	C	A	T	A	T	T	C	.	A	.	G	.	A	.	C	T	A	C	A	C	C	.	.	C
KJ617364	*Katsuwonus pelamis*	C	T	T	T	C	T	C	.	C	.	T	C	A	T	A	T	T	C	.	A	.	G	.	A	.	C	T	A	C	A	C	C	.	.	C
KJ617363	*Katsuwonus pelamis*	C	T	T	T	C	T	C	.	C	.	T	C	A	T	A	T	T	C	.	A	.	G	A	A	.	C	T	A	C	A	C	C	.	.	C
KJ617362	*Katsuwonus pelamis*	C	T	T	T	C	T	C	.	C	.	T	C	A	T	A	T	T	C	.	A	.	G	.	A	.	C	T	A	C	A	C	C	G	.	C
KJ617361	*Katsuwonus pelamis*	C	.	T	T	C	T	C	.	C	.	T	C	A	T	A	T	T	C	.	A	.	G	.	A	.	C	T	A	C	A	C	C	G	.	C
KJ617360	*Katsuwonus pelamis*	C	T	T	T	C	T	C	.	C	.	T	C	A	T	A	T	T	C	.	A	.	G	.	A	.	C	T	A	C	A	C	C	.	.	C
KJ617359	*Katsuwonus pelamis*	C	T	T	T	C	T	C	.	C	.	T	C	A	T	A	T	T	C	.	A	.	G	.	A	.	C	T	A	C	A	C	C	.	.	C
KJ617358	*Katsuwonus pelamis*	C	T	T	T	C	T	C	.	C	.	T	C	A	T	A	T	T	C	.	A	.	G	.	A	.	C	T	A	C	A	C	C	.	.	C
KJ617357	*Katsuwonus pelamis*	C	T	T	T	C	T	C	.	C	.	T	C	A	T	A	T	T	C	.	A	.	G	.	A	.	C	T	A	C	A	C	C	.	.	C
KJ617356	*Katsuwonus pelamis*	C	T	T	T	C	T	C	.	C	.	T	C	A	T	A	T	T	C	.	A	.	G	.	A	.	C	T	A	C	A	C	C	.	.	C
KJ617355	*Katsuwonus pelamis*	C	.	T	T	C	T	C	.	C	.	T	C	A	T	A	T	T	C	.	A	.	G	.	A	.	C	T	G	C	A	C	C	.	.	C
KJ617354	*Katsuwonus pelamis*	C	T	T	T	C	T	C	.	C	.	T	C	A	T	A	T	T	C	.	A	.	G	.	A	.	C	T	A	C	A	C	C	.	.	C

Supplementary Material

		2	6	9	18	21	30	33	36	39	45	63	69	72	75	84	93	96	102	105	111	120	123	129	132	144	156	162	174	180	183	192	198	213	219	231
KJ617353	*Katsuwonus pelamis*	C	T	T	T	C	T	C	.	C	.	T	C	A	T	A	T	T	C	.	A	.	G	.	A	.	C	T	A	C	A	C	C	G	.	C
KJ617352	*Katsuwonus pelamis*	C	T	T	T	C	T	C	.	C	.	T	C	A	T	A	T	T	C	.	A	.	G	.	A	.	C	T	A	C	A	C	C	.	.	C
KJ617351	*Katsuwonus pelamis*	C	T	T	T	C	T	C	.	C	.	T	C	A	T	A	T	T	C	.	A	.	G	.	A	.	C	.	A	C	A	C	C	.	.	C
KJ617350	*Katsuwonus pelamis*	C	T	T	T	C	T	C	.	C	.	T	C	A	T	A	T	T	C	.	A	.	G	.	A	.	C	T	A	C	A	C	C	.	.	C
KJ617349	*Katsuwonus pelamis*	C	T	T	T	C	T	C	.	C	.	T	C	A	T	A	T	T	C	.	A	.	G	.	A	.	C	T	A	C	A	C	C	.	.	C
KJ617348	*Katsuwonus pelamis*	C	T	T	T	C	T	C	.	C	.	T	C	A	T	A	T	T	C	.	A	.	G	.	A	.	C	T	A	C	A	C	C	.	.	C
KJ617347	*Katsuwonus pelamis*	C	T	T	T	C	T	C	.	C	.	T	C	A	T	A	T	T	C	.	A	.	G	.	A	.	C	T	A	C	A	C	C	.	.	C
KJ617346	*Katsuwonus pelamis*	C	T	T	T	C	T	C	.	C	.	T	C	A	T	A	T	T	C	.	A	.	G	.	A	.	C	T	A	C	A	C	C	.	.	C
KJ617345	*Katsuwonus pelamis*	C	T	T	T	C	T	C	.	C	.	T	C	A	T	A	T	T	C	.	A	.	G	.	A	.	C	T	A	C	A	C	C	.	.	C
KJ617344	*Katsuwonus pelamis*	C	T	T	T	C	T	C	.	C	.	T	C	A	T	A	T	T	C	.	A	.	G	.	A	.	C	T	A	C	A	C	C	G	.	C
KJ617343	*Katsuwonus pelamis*	C	T	T	T	C	T	C	.	C	.	T	C	A	T	A	T	T	C	.	A	.	G	.	A	.	C	T	A	C	A	C	C	.	.	C
KJ617342	*Katsuwonus pelamis*	C	T	T	T	C	T	C	.	C	.	T	C	A	T	A	T	T	C	.	A	.	.	.	A	.	C	T	A	C	A	C	C	.	.	C
KJ617341	*Katsuwonus pelamis*	C	T	T	T	C	T	C	.	C	.	T	C	A	T	A	T	T	C	.	A	.	.	.	A	.	C	T	A	C	A	C	C	.	.	C
KJ617340	*Katsuwonus pelamis*	C	T	T	T	C	T	C	.	C	.	T	C	A	T	A	T	T	C	.	A	.	.	.	A	.	C	T	A	C	A	C	C	.	.	C
KJ617339	*Katsuwonus pelamis*	C	T	T	T	C	T	C	.	C	.	T	C	A	T	A	T	T	C	.	A	.	G	.	A	.	C	T	A	C	A	C	C	.	.	C
KJ617338	*Katsuwonus pelamis*	C	T	T	T	C	T	C	.	C	.	T	C	A	T	A	T	T	C	.	A	.	G	.	A	.	C	T	A	C	A	C	C	G	.	C
KJ617337	*Katsuwonus pelamis*	C	T	T	T	C	T	C	.	C	.	T	C	A	T	A	T	T	C	.	A	.	G	.	A	.	C	T	A	C	A	C	C	.	.	C
KJ617336	*Katsuwonus pelamis*	C	T	T	T	C	T	C	.	C	.	T	C	A	T	A	T	T	C	.	A	.	G	.	A	.	C	T	A	C	A	C	C	.	.	C
KJ617335	*Katsuwonus pelamis*	C	T	T	T	C	T	C	.	C	.	T	C	A	T	A	T	T	C	.	A	.	G	.	A	.	C	T	A	C	A	C	C	.	.	C
KJ617334	*Katsuwonus pelamis*	C	T	T	T	C	T	C	.	C	.	T	C	A	T	A	T	T	C	.	A	.	G	.	A	.	C	T	A	C	A	C	C	.	.	C
KJ617333	*Katsuwonus pelamis*	C	T	T	T	C	T	C	.	C	.	T	C	A	T	A	T	T	C	.	A	.	G	.	A	.	C	T	A	C	A	C	C	.	.	C
KJ617332	*Katsuwonus pelamis*	C	T	T	T	C	T	C	.	C	.	T	C	A	T	A	T	T	C	.	A	.	G	.	A	.	C	T	A	C	A	C	C	.	.	C
KJ617331	*Katsuwonus pelamis*	C	T	T	T	C	T	C	.	C	.	T	C	A	T	A	T	T	C	.	A	.	G	.	A	.	C	T	A	C	A	C	C	.	.	C
KJ617330	*Katsuwonus pelamis*	C	T	T	T	C	T	C	.	C	.	T	C	A	T	A	T	T	C	.	A	.	G	.	A	.	C	T	A	C	A	C	C	.	.	C
KJ617329	*Katsuwonus pelamis*	C	T	T	T	C	T	C	.	C	.	T	C	G	T	A	T	T	C	.	A	.	G	.	A	.	C	T	A	C	A	C	C	.	.	C
KJ617328	*Katsuwonus pelamis*	C	T	T	T	C	T	C	.	C	.	T	C	A	T	A	T	T	C	.	A	.	G	.	A	.	C	T	A	C	A	C	C	.	.	C
KJ617327	*Katsuwonus pelamis*	C	T	T	T	C	T	C	.	C	.	T	C	A	T	A	T	T	C	.	A	.	G	.	A	.	C	T	A	C	A	C	C	G	C	C
KJ617326	*Katsuwonus pelamis*	C	T	T	T	C	T	C	.	C	.	T	C	A	T	A	T	T	C	.	A	.	G	.	A	.	C	T	A	C	A	C	C	G	.	C
KJ617325	*Katsuwonus pelamis*	C	T	T	T	C	T	C	.	C	.	T	C	A	T	A	T	T	C	.	A	.	G	.	A	.	C	T	A	C	A	C	C	G	.	C
KJ617324	*Katsuwonus pelamis*	C	T	T	T	C	T	C	.	C	.	T	C	A	T	A	T	T	C	.	A	.	G	.	A	.	C	T	A	C	A	C	C	.	.	C
KJ617323	*Katsuwonus pelamis*	C	T	T	T	C	T	C	.	C	.	T	C	A	T	A	T	T	C	.	A	.	G	.	A	.	C	T	A	C	A	C	C	G	.	C
KJ617322	*Katsuwonus pelamis*	C	T	T	T	C	T	C	.	C	.	T	C	A	T	A	T	T	C	.	A	.	G	.	A	.	C	T	A	C	A	C	C	G	.	C
KJ617321	*Katsuwonus pelamis*	C	T	T	T	C	T	C	.	C	.	T	C	A	T	A	T	T	C	.	A	.	G	.	A	.	C	T	A	C	A	C	C	.	.	C
KJ617320	*Katsuwonus pelamis*	C	T	T	T	C	T	C	.	C	.	T	C	A	T	A	T	T	C	.	A	.	G	.	A	.	C	T	A	C	A	C	C	.	.	C
KJ617319	*Katsuwonus pelamis*	C	T	T	T	C	T	C	.	C	.	T	C	A	T	A	T	T	C	.	A	.	.	.	A	.	C	T	A	C	A	C	C	.	.	C
KJ617318	*Katsuwonus pelamis*	C	T	T	T	C	T	C	.	C	.	T	C	A	T	A	T	T	C	T	A	.	G	.	A	.	C	T	A	C	A	C	C	.	.	C
KJ617317	*Katsuwonus pelamis*	C	T	T	T	C	T	C	.	C	.	T	C	A	T	A	T	T	C	.	A	.	G	.	A	.	C	T	A	C	A	C	C	.	.	C
KJ617316	*Katsuwonus pelamis*	C	.	T	T	C	T	C	.	C	.	T	C	A	T	A	T	T	C	.	A	.	G	.	A	.	C	T	A	C	A	C	C	.	.	C
KJ617315	*Katsuwonus pelamis*	C	T	T	T	C	T	C	.	C	.	T	C	A	T	A	T	T	C	.	A	.	G	.	A	.	C	T	A	C	A	C	C	.	.	C
KJ617314	*Katsuwonus pelamis*	C	T	T	T	C	T	C	.	C	.	T	C	A	T	A	T	T	C	.	A	.	G	.	A	.	C	T	A	C	A	C	C	.	.	C
KJ617313	*Katsuwonus pelamis*	C	T	T	T	C	T	C	.	C	.	T	C	A	T	A	T	T	C	.	A	.	G	.	A	.	C	T	A	C	A	C	C	.	.	C
KJ617312	*Katsuwonus pelamis*	C	T	T	T	C	T	C	.	C	.	T	C	A	T	A	T	T	C	.	A	.	G	.	A	.	C	T	A	C	A	C	C	.	.	C

Supplementary Material

		2	6	9	18	21	30	33	36	39	45	63	69	72	75	84	93	96	102	105	111	120	123	129	132	144	156	162	174	180	183	192	198	213	219	231
KJ617311	*Katsuwonus pelamis*	C	T	T	T	C	T	C	.	C	.	T	C	A	T	A	T	T	C	.	A	.	G	.	A	.	C	T	A	C	A	C	C	.	.	C
KJ617310	*Katsuwonus pelamis*	C	T	T	T	C	T	C	.	C	.	T	C	A	T	A	T	T	C	.	A	.	.	.	A	.	C	T	A	C	A	C	C	.	.	C
KJ617309	*Katsuwonus pelamis*	C	T	T	T	C	T	C	.	C	.	T	C	A	T	A	T	T	C	.	A	.	.	.	A	.	C	T	A	C	A	C	C	.	.	C
KJ617308	*Katsuwonus pelamis*	C	T	T	T	C	T	C	.	C	.	T	C	A	T	A	T	T	C	.	A	.	.	.	A	.	C	T	A	C	A	C	C	.	.	C
KJ617307	*Katsuwonus pelamis*	C	T	T	T	C	T	C	.	C	.	T	C	A	T	A	T	T	C	.	A	.	.	.	A	.	C	T	A	C	A	C	C	.	.	C
KJ617306	*Katsuwonus pelamis*	C	T	T	T	C	.	C	.	C	.	T	C	A	T	A	T	T	C	.	A	.	.	.	A	.	C	T	A	C	A	C	C	.	.	C
KJ617305	*Katsuwonus pelamis*	C	T	T	T	C	T	C	.	C	.	T	C	A	T	A	T	T	C	.	A	.	.	.	A	.	C	T	A	C	A	C	C	.	.	C
KJ617304	*Katsuwonus pelamis*	C	T	T	T	C	T	C	.	C	.	T	C	A	T	A	T	T	C	.	A	.	.	.	A	.	C	T	A	C	A	C	C	.	.	C
KJ617303	*Katsuwonus pelamis*	C	T	T	T	C	.	A	.	C	.	T	C	A	T	A	T	T	C	.	A	.	.	.	A	.	C	T	A	C	A	C	C	G	.	C
KJ617302	*Katsuwonus pelamis*	C	T	T	T	C	T	A	.	C	.	T	T	A	T	A	T	T	C	.	A	.	.	.	A	.	C	T	A	C	A	C	C	.	.	C
KJ617301	*Katsuwonus pelamis*	C	T	T	T	C	T	C	.	C	.	T	C	A	T	A	T	T	C	.	A	.	.	.	A	.	C	T	A	C	A	C	C	G	.	C
KJ617300	*Katsuwonus pelamis*	C	T	T	T	C	T	C	.	C	.	T	C	A	T	A	T	T	C	.	A	.	.	.	A	.	C	T	A	C	A	C	C	G	.	C
KJ617299	*Katsuwonus pelamis*	C	T	T	T	C	T	C	.	C	.	T	C	A	T	A	T	T	C	T	A	.	.	.	A	.	C	T	A	C	A	C	C	.	.	C
KJ617298	*Katsuwonus pelamis*	C	T	T	T	C	T	C	.	C	.	T	C	A	T	A	T	T	C	T	A	.	.	.	A	.	C	T	A	C	A	C	C	.	C	C
KJ617297	*Katsuwonus pelamis*	C	T	T	T	C	.	C	.	C	.	T	C	A	T	A	T	T	C	T	A	.	.	.	A	.	C	T	A	C	A	C	C	.	.	C
KJ617296	*Katsuwonus pelamis*	C	T	T	T	C	T	C	.	C	.	T	C	A	T	A	T	T	C	T	A	.	.	.	A	.	C	T	A	C	A	C	C	.	.	.
KJ617295	*Katsuwonus pelamis*	C	T	T	T	C	T	C	.	C	.	T	C	A	T	A	T	T	C	T	A	.	.	.	A	.	C	T	A	C	A	C	C	G	.	C
KJ617294	*Katsuwonus pelamis*	C	T	T	T	C	T	C	.	C	.	T	C	A	T	A	T	T	C	T	A	.	.	.	A	.	C	T	G	C	A	C	C	G	.	C
KJ617293	*Katsuwonus pelamis*	C	T	T	T	C	T	C	.	C	.	T	C	A	T	A	T	T	C	T	A	.	.	.	A	.	C	T	G	C	A	C	C	.	.	C
KJ617292	*Katsuwonus pelamis*	C	T	T	T	C	T	C	.	C	.	T	C	A	T	A	T	T	C	T	A	.	.	.	A	.	C	T	A	C	A	C	C	.	.	C
KJ617291	*Katsuwonus pelamis*	C	T	T	T	C	T	C	.	C	.	T	C	A	T	A	T	T	C	T	A	.	.	.	A	.	C	T	A	C	A	C	C	.	.	C
KJ617290	*Katsuwonus pelamis*	C	T	T	T	C	T	C	.	C	.	T	C	A	T	A	T	T	C	T	A	.	.	.	A	.	C	T	A	C	A	C	C	.	.	C
KJ617289	*Katsuwonus pelamis*	C	T	T	T	C	T	C	.	C	.	T	C	A	T	A	T	T	C	T	A	.	.	.	A	.	C	T	A	C	A	C	C	.	.	C
KJ617288	*Katsuwonus pelamis*	C	T	T	T	C	T	C	.	C	.	T	C	A	T	A	T	T	C	T	A	.	.	.	A	.	C	T	A	C	A	C	C	.	.	C
KJ617287	*Katsuwonus pelamis*	C	T	T	T	C	T	C	.	C	.	T	C	A	T	A	T	T	C	T	A	.	.	.	A	.	C	T	A	C	A	C	C	G	.	C
KJ617286	*Katsuwonus pelamis*	C	T	T	.	C	T	C	.	.	.	T	C	A	T	A	T	T	C	T	A	.	.	.	A	.	C	T	A	C	A	C	C	G	C	C
KJ617285	*Katsuwonus pelamis*	C	T	T	.	C	T	C	.	C	.	T	C	A	T	A	T	T	C	T	A	.	.	.	A	.	C	T	A	C	A	C	C	G	.	C
KJ617284	*Katsuwonus pelamis*	C	T	T	.	C	T	C	.	C	.	T	C	A	T	A	T	T	C	T	A	.	.	.	G	.	C	T	A	C	A	C	C	G	.	C
KJ617283	*Katsuwonus pelamis*	C	T	T	T	C	T	C	.	C	.	T	C	A	C	A	T	T	C	T	A	.	.	.	A	.	C	T	A	C	A	C	C	G	.	C
KJ617282	*Katsuwonus pelamis*	C	T	T	T	C	T	C	.	C	.	T	C	A	C	A	T	T	C	T	A	.	.	.	A	.	C	T	A	C	A	C	C	G	.	C
KJ617281	*Katsuwonus pelamis*	C	T	T	T	C	T	C	.	C	.	T	C	A	T	A	T	T	C	T	A	.	.	.	A	.	C	T	A	C	A	C	C	G	.	C
KJ617280	*Katsuwonus pelamis*	C	T	T	T	C	T	C	.	C	.	T	C	A	T	A	T	T	C	T	A	.	.	.	A	.	C	T	A	C	A	C	C	G	.	C
KJ617279	*Katsuwonus pelamis*	C	T	T	T	C	T	C	.	C	.	T	C	A	T	A	T	T	C	T	A	.	.	.	A	.	C	T	A	C	A	C	C	G	.	C
KJ617278	*Katsuwonus pelamis*	C	.	T	C	C	T	C	.	C	.	T	C	A	T	A	T	T	C	T	A	.	.	.	A	.	C	T	A	C	A	C	C	G	.	C
KJ617277	*Katsuwonus pelamis*	C	T	T	T	C	T	C	.	C	.	T	C	A	T	A	T	T	C	T	A	.	.	.	A	.	C	T	A	C	A	C	C	G	.	C
KJ617276	*Katsuwonus pelamis*	C	T	T	T	C	T	C	.	C	.	T	C	A	T	A	T	T	C	T	A	.	.	.	A	.	C	T	A	C	A	C	C	G	.	C
KJ617275	*Katsuwonus pelamis*	C	T	T	T	C	T	C	.	C	.	T	C	A	T	A	T	T	C	T	A	.	.	.	A	.	C	T	A	C	A	C	C	G	.	C
KJ617274	*Katsuwonus pelamis*	C	T	T	T	C	T	C	.	C	.	T	C	A	T	A	T	T	C	T	A	.	.	.	A	.	C	T	A	C	A	C	C	G	.	C
KJ617273	*Katsuwonus pelamis*	C	T	T	T	C	T	C	.	C	.	T	C	A	T	A	T	T	C	T	A	.	.	.	A	.	C	T	A	C	A	C	C	G	.	C
KJ617272	*Katsuwonus pelamis*	C	T	T	T	C	T	C	.	C	.	T	C	A	T	A	T	T	C	T	A	.	.	.	A	.	C	T	A	C	A	C	C	G	.	C
KJ617271	*Katsuwonus pelamis*	C	T	T	T	C	T	C	.	C	.	T	C	A	T	A	T	T	C	T	A	.	.	.	A	.	C	T	A	C	A	C	C	G	.	C
KJ617270	*Katsuwonus pelamis*	C	T	T	T	C	.	C	.	C	.	T	C	A	T	A	T	T	C	T	A	.	.	.	A	.	C	T	A	C	A	C	C	.	.	C

Supplementary Material

		2	6	9	18	21	30	33	36	39	45	63	69	72	75	84	93	96	102	105	111	120	123	129	132	144	156	162	174	180	183	192	198	213	219	231
KJ617269	*Katsuwonus pelamis*	C	T	T	T	C	T	C	.	C	.	T	C	A	T	A	T	T	C	T	A	.	.	.	A	.	C	T	A	C	A	C	C	.	.	C
KJ617268	*Katsuwonus pelamis*	C	T	T	T	C	T	C	.	C	.	T	C	A	T	A	T	T	C	T	A	.	.	.	A	.	C	T	A	C	A	C	C	.	.	C
KJ617267	*Katsuwonus pelamis*	C	T	T	T	C	T	C	.	C	.	T	C	A	T	A	T	T	C	T	A	.	.	.	A	.	C	T	A	C	A	C	C	G	.	C
KJ617266	*Katsuwonus pelamis*	C	T	T	T	C	T	C	.	C	.	T	C	A	T	A	T	T	C	T	A	.	.	.	A	.	C	T	A	C	A	C	C	G	.	C
KJ617265	*Katsuwonus pelamis*	C	T	T	T	C	T	C	.	C	.	T	C	A	T	A	T	T	C	T	A	.	.	.	A	.	C	T	A	C	A	C	C	.	.	C
KJ617264	*Katsuwonus pelamis*	C	T	T	T	C	T	C	.	.	.	T	C	A	T	A	T	T	C	T	A	.	.	.	A	.	C	T	A	C	A	C	C	G	.	C
KJ617263	*Katsuwonus pelamis*	C	T	T	T	C	T	C	.	C	.	T	C	A	T	A	T	T	C	T	A	.	.	.	A	.	C	T	A	C	A	C	C	.	.	C
KJ617262	*Katsuwonus pelamis*	C	T	T	T	C	T	C	.	C	.	T	C	A	T	A	T	T	C	T	A	.	.	.	A	.	C	T	A	C	A	C	C	G	.	C
KJ617261	*Katsuwonus pelamis*	C	T	T	T	C	T	C	.	C	.	T	C	A	T	A	T	T	C	T	A	.	.	.	A	.	C	T	A	C	A	C	C	G	.	C
KJ617260	*Katsuwonus pelamis*	C	T	T	.	C	T	C	.	C	.	T	C	A	T	A	T	T	C	T	A	.	.	.	A	.	C	T	A	C	A	C	C	G	.	C
KJ617259	*Katsuwonus pelamis*	C	T	T	T	C	T	C	.	C	.	T	C	A	T	A	T	T	C	T	A	.	.	.	A	.	C	T	A	C	A	C	C	G	.	C
KJ617258	*Katsuwonus pelamis*	C	T	T	T	C	T	C	.	C	.	T	C	A	T	A	T	T	C	T	A	.	.	.	A	.	C	T	A	C	A	C	C	G	.	C
DQ497840	*Katsuwonus pelamis*	C	T	T	T	C	T	C	.	C	.	T	C	A	T	A	T	T	C	T	A	.	.	.	A	.	C	T	A	C	A	C	C	.	.	C
DQ497839	*Katsuwonus pelamis*	C	T	T	T	C	T	C	.	C	.	T	C	A	T	A	T	T	C	T	A	.	.	.	A	.	C	T	A	C	A	C	C	.	.	C
DQ497838	*Katsuwonus pelamis*	C	T	T	T	C	T	C	.	C	.	T	C	A	T	A	T	T	C	.	A	.	G	.	A	.	C	T	A	C	A	C	C	.	.	C
DQ080321	*Katsuwonus pelamis*	C	.	T	T	C	T	C	.	C	.	T	C	A	T	A	T	T	C	.	A	.	.	.	A	.	C	T	A	C	A	C	C	G	.	C
DQ080320	*Katsuwonus pelamis*	C	T	T	T	C	T	C	.	C	.	T	C	A	T	A	T	T	C	.	A	.	G	.	A	.	C	T	A	C	A	C	C	G	.	C
DQ080319	*Katsuwonus pelamis*	C	T	T	T	C	T	C	.	C	.	T	C	A	T	A	T	T	C	.	A	.	.	.	A	.	C	T	A	C	A	C	C	.	.	C
DQ080318	*Katsuwonus pelamis*	C	T	T	T	C	T	C	.	C	.	T	C	A	T	A	T	T	C	.	A	.	.	.	A	.	C	T	A	C	A	C	C	G	.	C
DQ080317	*Katsuwonus pelamis*	C	T	T	T	C	T	C	.	C	.	T	C	A	T	A	T	T	C	.	A	.	G	.	A	.	C	T	A	C	A	C	C	.	.	C
DQ080316	*Katsuwonus pelamis*	C	T	T	T	C	T	C	.	C	.	T	C	A	T	A	T	T	C	.	A	.	.	.	A	.	C	T	A	C	A	C	C	.	.	C
DQ080315	*Katsuwonus pelamis*	C	T	T	T	C	T	C	.	C	.	T	C	A	T	A	T	T	C	.	A	.	G	.	A	.	C	T	A	C	A	C	C	.	.	C
EU250983	*Katsuwonus pelamis*	C	T	T	T	C	T	C	.	C	.	T	C	A	T	A	T	T	C	.	A	.	G	.	A	.	C	T	A	C	A	C	C	.	.	C
EU250981	*Katsuwonus pelamis*	C	T	T	T	C	T	C	.	C	.	T	C	A	T	A	T	T	C	.	A	.	G	.	A	.	C	.	A	C	A	C	C	.	.	C
KU904414	*Katsuwonus pelamis*	C	T	T	T	C	T	C	.	C	.	T	C	A	T	A	T	T	C	.	A	.	.	.	A	.	C	T	A	C	A	C	C	.	.	C
EU250982	*Katsuwonus pelamis*	C	T	T	T	C	T	C	.	C	.	T	C	A	T	A	T	T	C	.	A	.	G	.	A	.	C	T	A	C	A	C	C	.	.	C
EU250975	*Katsuwonus pelamis*	C	T	T	T	C	T	C	.	C	.	T	C	A	T	A	T	T	C	T	A	.	.	.	A	.	C	T	A	C	A	C	C	G	.	C
EU250974	*Katsuwonus pelamis*	C	T	T	T	C	T	C	.	C	.	T	C	A	T	A	T	T	C	.	A	.	G	.	A	.	C	T	A	C	A	C	C	G	.	C
EU250976	*Katsuwonus pelamis*	C	.	T	T	C	T	C	.	C	.	T	C	A	T	A	T	T	C	.	A	.	G	.	A	.	C	T	G	C	A	C	C	.	.	C
EU250967	*Katsuwonus pelamis*	C	T	T	T	C	T	C	.	C	.	T	C	A	T	A	T	T	C	T	A	.	.	.	A	.	C	T	A	C	A	C	C	G	.	C
AB101290	*Katsuwonus pelamis*	C	.	T	T	C	T	C	.	C	.	T	C	A	T	A	T	T	C	T	A	.	.	.	A	.	C	T	A	C	A	C	C	.	.	C
EU250966	*Katsuwonus pelamis*	C	T	T	T	.	T	C	.	C	.	T	C	A	T	A	T	T	C	.	A	.	G	.	A	.	C	T	A	C	A	C	C	.	.	C
NC 005316	*Katsuwonus pelamis*	C	.	T	T	C	T	C	.	C	.	T	C	A	T	A	T	T	C	T	A	.	.	.	A	.	C	T	A	C	A	C	C	.	.	C
GU256527	*Katsuwonus pelamis*	C	T	T	T	C	T	C	.	C	.	T	C	A	T	A	T	T	C	T	A	.	.	.	A	.	C	T	A	C	A	C	C	.	.	C
EF141175	*Katsuwonus pelamis*	C	T	T	T	C	T	C	.	C	.	T	C	A	T	A	T	T	C	.	A	.	G	.	A	.	C	T	A	C	A	C	C	.	.	C
JN086155	*Katsuwonus pelamis*	C	T	T	T	C	T	C	.	C	.	T	C	A	T	A	T	T	C	T	A	.	.	.	A	.	C	T	A	C	A	C	C	.	.	C
KM605252	*Katsuwonus pelamis*	C	T	T	T	C	T	C	.	C	.	T	C	A	T	A	T	T	C	.	A	.	.	.	A	.	C	T	A	C	A	C	C	G	.	C
	SKJ-AO-L1-1	C	T	T	T	C	T	C	.	C	.	T	C	A	T	A	T	T	C	T	A	.	.	.	A	.	C	T	A	C	A	C	C	G	C	C
	SKJ-AO-L1-2	C	T	T	T	C	T	C	.	C	.	T	C	A	T	A	T	T	C	.	A	.	.	.	A	.	C	T	A	C	A	C	C	.	C	C
	SKJ-AO-L1-3	C	T	T	T	C	T	C	.	C	.	T	C	A	C	A	T	T	C	T	A	.	.	.	A	.	C	T	A	C	A	C	C	G	C	C
	SKJ-IO-L1-1	C	T	T	T	C	T	C	.	C	.	T	C	A	T	A	T	T	C	.	A	.	G	.	A	.	C	T	A	C	A	C	C	G	C	C
	SKJ-IO-L1-2	C	T	T	T	C	T	C	.	C	.	T	C	A	T	A	T	T	C	T	A	.	.	.	A	.	C	T	A	C	A	C	C	G	C	C

Supplementary Material

		2	6	9	18	21	30	33	36	39	45	63	69	72	75	84	93	96	102	105	111	120	123	129	132	144	156	162	174	180	183	192	198	213	219	231
	SKJ-IO-L1-3	C	T	T	T	C	T	C	.	C	.	T	C	A	T	A	T	T	C	.	A	.	G	.	A	.	C	T	A	C	A	C	C	G	.	C
	SKJ-WCPO-L1-1	C	T	T	T	C	T	C	.	C	.	T	C	A	T	A	T	T	C	T	A	.	.	.	A	.	C	T	A	C	A	C	C	G	C	C
	SKJ-WCPO-L1-2	C	T	T	T	C	T	C	.	C	.	T	C	A	T	A	T	T	C	.	A	.	.	.	A	.	C	T	A	C	A	C	C	.	C	C
	SKJ-WCPO-L1-3	C	T	T	T	C	T	C	.	C	.	T	C	A	T	A	T	T	C	.	A	.	G	.	A	.	C	T	A	C	A	C	C	.	.	C
	SKJ-EPO-L1-1	C	T	T	T	C	T	C	.	C	.	T	C	A	T	A	T	T	C	T	A	.	.	.	A	.	C	T	A	C	A	C	C	.	C	C
	SKJ-EPO-L1-2	C	T	T	T	C	T	C	.	C	.	T	C	A	T	A	T	T	C	.	A	.	.	.	A	.	C	T	A	C	A	C	C	.	C	C
	SKJ-EPO-L1-3	C	T	T	T	C	T	C	.	C	.	T	C	A	T	A	T	T	C	.	A	.	G	.	A	.	C	T	A	C	A	C	C	.	C	C
MG017702	*Thunnus tonggol*	.	.	.	.	.	.	.	T	.	.	.	.	.	T	.	.	.	.	.	.	G	.	.	.	.	.	T	.	.	.	.	.	.	.	.
DQ497920	*Thunnus tonggol*	.	.	.	.	.	.	.	T	.	.	.	.	.	T	.	.	.	.	.	.	G	.	.	.	.	.	T	.	.	.	.	.	.	.	.
DQ497919	*Thunnus tonggol*	.	.	.	.	.	.	.	T	.	.	.	.	.	T	.	.	.	.	.	.	G	.	.	.	.	.	T	.	.	.	.	.	.	.	.
DQ497918	*Thunnus tonggol*	.	.	.	.	.	.	.	T	.	.	.	.	.	T	.	.	.	.	.	.	G	.	A	.	.	.	T	.	.	.	.	.	.	.	.
DQ497917	*Thunnus tonggol*	.	.	.	.	.	.	.	T	.	.	.	.	.	T	.	.	.	.	.	.	G	.	A	.	.	.	T	.	.	.	.	.	.	.	.
DQ497916	*Thunnus tonggol*	.	.	.	.	.	.	.	T	.	.	.	.	.	T	.	.	.	.	.	.	G	.	.	.	.	.	T	.	.	.	.	C	.	.	.
DQ497915	*Thunnus tonggol*	.	.	.	.	.	.	.	T	.	.	.	.	.	T	.	.	.	.	.	.	G	.	.	.	.	.	T	.	.	.	.	.	.	.	.
EU935788	*Thunnus tonggol*	.	.	.	.	.	.	.	T	.	.	.	.	.	T	.	.	.	.	.	.	G	.	.	.	.	.	T	.	.	.	.	.	.	.	.
EU935787	*Thunnus tonggol*	.	.	.	.	.	.	.	T	.	.	.	.	.	T	.	.	.	.	.	.	G	.	.	.	.	.	T	.	.	.	.	.	.	.	.
EU935786	*Thunnus tonggol*	.	.	.	.	.	.	.	T	.	.	.	.	.	T	.	.	.	.	.	.	G	.	.	.	.	.	T	.	.	.	.	.	.	.	.
EU935785	*Thunnus tonggol*	.	.	.	.	.	.	.	T	.	.	.	.	.	T	.	.	.	.	.	.	G	.	.	.	.	.	T	.	.	.	.	.	.	.	.
EU349415	*Thunnus tonggol*	.	.	.	.	.	.	.	T	.	.	.	.	.	T	.	.	.	.	.	.	G	.	.	.	.	.	T	.	.	.	.	.	.	.	.
EU349414	*Thunnus tonggol*	.	.	.	.	.	.	.	T	.	.	.	.	.	T	.	.	.	.	.	.	G	.	.	.	.	.	T	.	.	.	.	.	.	.	.
EU349413	*Thunnus tonggol*	.	.	.	.	.	.	.	T	.	.	.	.	.	T	.	.	.	.	.	.	G	.	.	.	.	.	T	.	.	.	.	.	.	.	.
DQ497914	*Thunnus tonggol*	.	.	.	.	.	.	.	T	.	.	.	.	.	T	.	.	.	.	.	.	G	.	.	.	.	.	T	.	.	.	.	.	.	.	.
DQ497913	*Thunnus tonggol*	.	.	.	.	.	.	.	T	.	.	.	.	.	T	.	.	.	.	.	.	G	.	.	.	.	.	T	.	.	.	.	.	.	.	.
EF141181	*Thunnus tonggol*	.	.	.	.	.	.	.	T	.	.	.	.	.	T	.	.	.	.	.	.	G	.	.	.	.	.	T	.	.	.	.	.	.	.	.
KM055411	*Thunnus tonggol*	.	.	.	.	.	.	.	T	.	.	.	.	.	T	.	.	.	.	.	.	G	.	.	.	.	.	T	.	.	.	.	.	.	.	.
EU349412	*Thunnus tonggol*	.	.	.	.	.	.	.	T	.	.	.	.	.	T	.	.	.	.	.	.	G	.	.	.	.	.	T	.	.	.	.	.	.	.	.
EU935790	*Thunnus tonggol*	.	.	.	.	.	.	.	T	.	.	.	.	.	T	.	.	.	.	.	.	G	.	.	.	.	.	T	.	.	.	.	.	.	.	.
EU935789	*Thunnus tonggol*	.	.	.	.	.	.	.	T	.	.	.	.	.	T	.	.	.	.	.	.	G	.	.	.	.	.	T	.	.	.	.	.	.	.	.
KC522359	*Thunnus tonggol*	.	.	.	C	.	.	.	.	.	.	.	.	.	A	.	.	.	.	.	.	.	.	.	.	.	.	T	.	.	.	C	.	.	.	C
HQ425780	*Thunnus tonggol*	.	.	.	C	.	.	.	.	.	.	.	.	.	A	.	.	.	.	.	.	.	.	.	.	.	.	T	.	.	.	C	.	.	.	.
JN086154	*Thunnus tonggol*	.	.	.	C	.	.	.	.	.	.	.	.	.	A	.	.	.	.	.	.	.	.	.	.	.	.	T	.	.	.	C	.	.	.	.
NC 020673	*Thunnus tonggol*	.	.	.	C	.	.	.	.	.	.	.	.	.	A	.	.	.	.	.	.	.	.	.	.	.	.	T	.	.	.	C	.	.	.	.
	LOT-IO-L1-1	.	.	.	.	.	.	.	.	.	.	.	.	.	T	.	.	.	.	.	.	G	.	.	.	.	.	T	.	.	.	.	.	.	C	C
	LOT-IO-L1-2	.	.	.	.	.	.	.	.	.	.	.	.	.	T	.	.	.	.	.	.	G	.	.	.	.	.	T	.	.	.	.	.	.	C	C
	LOT-IO-L1-3	.	.	.	.	.	T	.	.	.	.	.	.	.	T	.	.	.	.	.	.	G	.	.	.	.	.	T	.	.	.	.	.	.	C	C
