# Supplementary material for: Canning Processes Reduce the DNA-Based Traceability of Commercial Tropical Tunas
Source: Foods. 2020 Sep 27;9(10):1372. doi: 10.3390/foods9101372 (PMC7650566; doi:10.3390/foods9101372)
Supplement: Supplementary file 1 [file foods-09-01372-s001.zip › SUPPLEMENTARY/Table S2_foods-943909_rev18_09_2020.pdf]

Supplementary Material

Pecoraro et al. Canning processes reduce DNA-based traceability of commercial tropical tunas

Table S2: Nucleotide substitution within the Cyt b sequences of the four target species (YFT, yellowfin tuna, *Thunnus albacares*; BET, bigeye tuna, *Thunnus obesus*; SKJ, skipjack tuna, *Katsuwonus pelamis*; LOT, longtail tuna, *Thunnus tonggol*) aligned against to the sequence of the mitochondrial genome of *Thunnus thynnus* (NCBI: NC_004901, positions 14665 – 14901, 236 bp). Sequences from other levels (L2-L4) were compared with the reference level (L1). Nucleotide substitution was calculated for each specimen as a percentage of the variable positions.

	2	6	9	18	21	30	33	36	39	45	63	69	72	75	84	93	96	102	105	111	120	123	129	132	144	156	162	174	180	183	192	198	213	219	222	231
NC 004901 T.thynnus	T	C	C	A	T	C	T	C	A	A	A	A	C	G	T	C	C	T	C	C	A	A	G	T	T	T	C	C	T	C	T	T	A	T	A	T
YFT-AO-L1-1	.	.	.	.	.	.	.	.	.	G	.	.	.	A	.	.	.	.	.	.	.	.	.	.	.	.	T	.	.	T	.	.	.	C	.	C
YFT-AO-L2-1	.	.	.	.	.	.	.	.	.	G	.	.	.	A	.	.	.	.	.	.	.	.	.	.	.	.	T	.	.	T	.	.	.	C	.	C
YFT-AO-L3-1	.	.	.	.	.	.	.	.	.	G	.	.	A	A	.	.	.	.	.	.	.	.	.	.	.	.	T	.	.	T	.	.	.	C	.	C
YFT-AO-L4O-1	-	-	-	-	-	-	-	-	-	-	-	-	-	-	-	-	-	-	-	-	-	-	.	.	.	.	T	.	.	.	C	.	.	C	.	C
YFT-AO-L4B-1	-	-	-	-	-	-	-	-	-	-	-	-	-	-	-	-	-	-	-	-	-	-	.	A	.	C	T	A	C	A	C	C	.	C	.	C
YFT-AO-L1-2	.	.	.	.	.	.	.	.	.	G	.	.	.	A	.	.	.	.	.	.	.	.	.	.	.	.	T	.	.	T	.	.	.	C	.	C
YFT-AO-L2-2	.	.	.	.	.	.	.	.	.	G	.	.	.	A	.	.	.	.	.	.	.	.	.	.	.	.	T	.	.	T	.	.	.	C	.	C
YFT-AO-L3-2	.	.	.	.	.	.	.	.	.	G	.	.	.	A	.	.	.	.	.	.	.	.	.	.	.	.	T	.	.	T	.	.	.	C	.	C
YFT-AO-L4O-2	-	-	-	-	-	-	-	-	-	-	-	-	-	-	-	-	-	-	-	-	-	-	.	A	.	C	T	A	C	A	C	C	.	C	.	C
YFT-AO-L4B-2	-	-	-	-	-	-	-	-	-	-	-	-	-	-	-	-	-	-	-	-	-	-	.	A	.	C	T	A	C	A	C	C	.	C	.	C
YFT-AO-L1-3	.	.	.	.	.	.	.	.	.	G	.	.	A	A	.	.	.	C	.	.	.	.	.	.	.	.	T	.	.	T	.	.	.	C	.	C
YFT-AO-L2-3	.	.	.	.	.	.	.	A	.	.	.	.	A	A	.	.	.	C	.	.	.	.	.	.	.	.	T	.	.	T	.	.	.	C	.	C
YFT-AO-L3-3	-	-	-	-	-	-	-	-	-	-	-	-	-	-	-	-	-	-	-	-	-	-	-	-	-	-	-	-	-	-	-	-	-	-	-	-
YFT-AO-L4O-3	-	-	-	-	-	-	-	-	-	-	-	-	-	-	-	-	-	-	-	-	-	-	.	.	.	.	T	.	.	T	.	.	.	.	.	C
YFT-AO-L4B-3	-	-	-	-	-	-	-	-	-	-	-	-	-	-	-	-	-	-	-	-	-	-	.	A	.	C	T	A	C	A	C	C	.	C	.	C
YFT-IO-L1-1	.	.	.	.	.	.	.	.	.	G	.	.	.	A	.	.	.	.	.	.	.	.	.	.	.	.	T	.	.	T	.	.	.	C	.	C
YFT-IO-L2-1	.	.	.	.	.	.	.	.	.	G	.	.	.	A	.	.	.	.	.	.	.	.	.	.	.	.	T	.	.	T	.	.	.	C	.	C
YFT-IO-L3-1	.	.	.	.	.	.	.	.	.	G	.	.	.	A	.	.	.	.	.	.	.	.	.	.	.	.	T	.	.	T	.	.	.	C	.	C
YFT-IO-L4O-1	-	-	-	-	-	-	-	-	-	-	-	-	-	-	-	-	-	-	-	-	-	-	.	A	.	C	T	A	.	T	C	C	.	.	.	C
YFT-IO-L4B-1	-	-	-	-	-	-	-	-	-	-	-	-	-	-	-	-	-	-	-	-	-	-	.	.	.	.	T	.	.	T	.	.	.	C	.	C
YFT-IO-L1-2	.	.	.	.	.	.	.	.	.	G	.	.	.	A	.	.	.	.	.	.	.	.	.	.	.	.	T	.	.	T	.	.	.	C	.	C
YFT-IO-L2-2	.	.	.	.	.	.	.	.	.	G	.	.	.	A	.	.	.	.	.	.	.	.	.	.	.	.	T	.	.	T	.	.	.	C	.	C
YFT-IO-L3-2	.	.	.	.	.	.	.	.	.	G	.	.	.	A	.	.	.	.	.	.	.	.	.	.	.	.	T	.	.	T	.	.	.	C	.	C
YFT-IO-L4O-2	-	-	-	-	-	-	-	-	-	-	-	-	-	-	-	-	-	-	-	-	-	-	.	.	.	.	T	.	.	T	.	.	.	C	.	C
YFT-IO-L4B-2	-	-	-	-	-	-	-	-	-	-	-	-	-	-	-	-	-	-	-	-	-	-	.	.	.	.	T	.	.	A	C	.	.	C	.	C
YFT-IO-L1-3	.	.	.	.	.	.	.	.	.	G	.	.	.	A	.	.	.	.	.	.	.	.	.	.	.	.	T	.	.	T	.	.	.	C	.	C
YFT-IO-L2-3	.	.	.	.	.	.	.	.	.	G	.	.	.	A	.	.	.	.	.	.	.	.	.	.	.	.	T	.	.	T	.	.	.	C	.	C
YFT-IO-L3-3	.	.	.	.	.	.	.	.	.	G	.	.	.	A	.	.	.	.	.	.	.	.	.	.	.	.	T	.	.	T	.	.	.	C	.	C
YFT-IO-L4O-3	-	-	-	-	-	-	-	-	-	-	-	-	-	-	-	-	-	-	-	-	-	-	.	.	.	.	T	.	.	T	.	.	.	.	.	C
YFT-IO-L4B-3	-	-	-	-	-	-	-	-	-	-	-	-	-	-	-	-	-	-	-	-	-	-	.	A	.	C	T	A	.	A	C	C	.	.	.	C

Supplementary Material

	2	6	9	18	21	30	33	36	39	45	63	69	72	75	84	93	96	102	105	111	120	123	129	132	144	156	162	174	180	183	192	198	213	219	222	231
NC 004901 T.thynnus	T	C	C	A	T	C	T	C	A	A	A	A	C	G	T	C	C	T	C	C	A	A	G	T	T	T	C	C	T	C	T	T	A	T	A	T
YFT-WCPO-L1-1	.	.	.	.	.	.	.	.	.	G	.	.	.	A	.	.	.	.	.	.	.	.	.	.	.	.	T	.	.	T	.	.	.	C	.	C
YFT-WCPO-L2-1	.	.	.	.	.	.	.	.	.	G	.	.	.	A	.	.	.	.	.	.	.	.	.	.	.	.	T	.	.	T	.	.	.	C	.	C
YFT-WCPO-L3-1	.	.	.	.	.	.	.	.	.	G	.	.	.	A	.	.	.	.	.	.	.	.	.	.	.	.	T	.	.	T	.	.	.	C	.	C
YFT-WCPO-L4O-1	-	-	-	-	-	-	-	-	-	-	-	-	-	-	-	-	-	-	-	-	-	-	.	.	.	.	T	.	.	T	.	.	.	C	.	C
YFT-WCPO-L4B-1	-	-	-	-	-	-	-	-	-	-	-	-	-	-	-	-	-	-	-	-	-	-	.	A	.	C	T	A	C	.	C	C	.	C	.	C
YFT-WCPO-L1-2	.	.	.	.	.	.	.	.	.	G	.	.	.	A	.	.	.	.	.	.	.	.	.	.	.	.	T	.	.	T	.	.	.	C	.	C
YFT-WCPO-L2-2	.	.	.	.	.	.	.	.	.	G	.	.	.	A	.	.	.	.	.	.	.	.	.	.	.	.	T	.	.	T	.	.	.	C	.	C
YFT-WCPO-L3-2	.	.	.	.	.	.	.	.	.	G	.	.	.	A	.	.	.	.	.	.	.	.	.	.	.	.	T	.	.	T	.	.	.	C	.	C
YFT-WCPO-L4O-2	-	-	-	-	-	-	-	-	-	-	-	-	-	-	-	-	-	-	-	-	-	-	.	.	.	.	T	.	.	T	.	.	.	C	.	C
YFT-WCPO-L4B-2	-	-	-	-	-	-	-	-	-	-	-	-	-	-	-	-	-	-	-	-	-	-	.	.	.	.	T	.	.	T	.	.	.	C	.	C
YFT-WCPO-L1-3	.	.	.	.	.	.	.	.	.	G	.	.	.	A	.	.	.	.	.	.	.	.	.	.	.	.	T	.	.	T	.	.	.	C	.	C
YFT-WCPO-L2-3	.	.	.	.	.	.	.	.	.	G	.	.	.	A	.	.	.	.	.	.	.	.	.	.	.	.	T	.	.	T	.	.	.	C	.	C
YFT-WCPO-L3-3	.	.	.	.	.	.	.	.	.	G	.	.	.	A	.	.	.	.	.	.	.	.	.	.	.	.	T	.	.	T	.	.	.	C	.	C
YFT-WCPO-L4O-3	-	-	-	-	-	-	-	-	-	-	-	-	-	-	-	-	-	-	-	-	-	-	.	.	.	.	T	.	.	T	.	.	.	.	.	C
YFT-WCPO-L4B-3	-	-	-	-	-	-	-	-	-	-	-	-	-	-	-	-	-	-	-	-	-	-	.	.	.	.	T	.	.	T	C	.	.	C	T	C
YFT-EPO-L1-1	.	.	.	.	.	.	.	.	.	G	.	.	.	A	.	.	.	.	.	.	.	.	.	.	C	.	T	.	.	T	.	.	.	C	.	C
YFT-EPO-L2-1	.	.	.	.	.	.	.	.	.	G	.	.	.	A	.	.	.	.	.	.	.	.	.	.	C	.	T	.	.	T	.	.	.	C	.	C
YFT-EPO-L3-1	.	.	.	.	.	.	.	.	.	G	.	.	.	A	.	.	.	.	.	.	.	.	.	.	C	.	T	.	.	T	.	.	.	C	.	C
YFT-EPO-L4O-1	-	-	-	-	-	-	-	-	-	-	-	-	-	-	-	-	-	-	-	-	-	-	.	.	C	.	T	.	.	T	.	.	.	C	.	C
YFT-EPO-L4B-1	-	-	-	-	-	-	-	-	-	-	-	-	-	-	-	-	-	-	-	-	-	-	.	A	.	.	T	A	.	T	C	.	.	C	.	C
YFT-EPO-L1-2	.	.	.	.	.	.	.	.	.	G	.	.	.	A	.	.	.	.	.	.	.	.	.	.	C	.	T	.	.	T	.	.	.	C	.	C
YFT-EPO-L2-2	.	.	.	.	.	.	.	.	.	G	.	.	.	A	.	.	.	.	.	.	.	.	.	.	C	.	T	.	.	T	.	.	.	C	.	C
YFT-EPO-L3-2	.	.	.	.	.	.	.	.	.	G	.	.	.	A	.	.	.	.	.	.	.	.	.	.	C	.	T	.	.	T	.	.	.	C	.	C
YFT-EPO-L4O-2	-	-	-	-	-	-	-	-	-	-	-	-	-	-	-	-	-	-	-	-	-	-	.	.	C	.	T	.	.	T	.	.	.	C	.	C
YFT-EPO-L4B-2	-	-	-	-	-	-	-	-	-	-	-	-	-	-	-	-	-	-	-	-	-	-	.	A	.	C	T	A	C	A	.	C	.	C	.	C
YFT-EPO-L1-3	.	.	.	.	.	.	.	.	.	G	.	.	.	A	.	.	.	.	.	.	.	.	.	.	C	.	T	.	.	T	.	.	.	C	.	C
YFT-EPO-L2-3	.	.	.	.	.	.	.	.	.	G	.	.	.	A	.	.	.	.	.	.	.	.	.	.	C	.	T	.	.	T	.	.	.	C	.	C
YFT-EPO-L3-3	.	.	.	.	.	.	.	.	.	G	.	.	.	A	.	.	.	.	.	.	.	.	.	.	C	.	T	.	.	T	.	.	.	C	.	C
YFT-EPO-L4O-3	-	-	-	-	-	-	-	-	-	-	-	-	-	-	-	-	-	-	-	-	-	-	.	.	C	.	T	.	.	T	.	.	.	C	.	C
YFT-EPO-L4B-3	-	-	-	-	-	-	-	-	-	-	-	-	-	-	-	-	-	-	-	-	-	-	.	.	.	C	T	.	.	T	C	.	.	C	.	C

Supplementary Material

	2	6	9	18	21	30	33	36	39	45	57	63	69	72	75	84	93	96	102	105	111	117	120	123	129	132	144	156	162	174	180	183	192	198	213	219	
NC 004901 T.thynnus	T	C	C	A	T	C	T	C	A	A	C	A	A	C	G	T	C	C	T	C	C	C	A	A	G	T	T	T	C	C	T	C	T	T	A	T	
BET-IO-L1-1	.	.	.	.	.	.	.	.	.	.	.	.	.	A	A	.	.	.	C	.	.	.	.	.	.	.	.	.	T	.	.	.	C	.	.	C	C
BET-IO-L2-1	.	.	.	.	.	.	.	.	.	.	.	.	.	A	A	.	.	.	C	.	.	.	.	.	.	.	.	.	T	.	.	.	C	.	.	C	C
BET-IO-L3-1	.	.	.	.	.	.	.	.	.	.	.	.	.	A	A	.	.	.	C	.	A	.	-	-	-	-	-	-	T	-	-	-	-	-	-	-	-
BET-IO-L4O-1	-	-	-	-	-	-	-	-	-	-	-	-	-	-	-	-	-	-	-	-	-	-	-	-	.	.	.	.	T	.	.	.	C	.	.	C	C
BET-IO-L4B-1	-	-	-	-	-	-	-	-	-	-	-	-	-	-	-	-	-	-	-	-	-	-	-	-	.	.	.	.	T	.	.	.	C	.	.	C	C
BET-IO-L1-2	.	.	.	.	.	.	.	.	.	.	.	.	.	A	A	.	.	.	C	.	.	.	.	.	.	.	.	.	T	.	.	.	C	.	.	C	C
BET-IO-L2-2	.	.	.	.	.	.	.	.	.	.	.	.	.	A	A	.	.	.	C	.	.	.	.	.	.	.	.	.	T	.	C	.	C	.	.	C	C
BET-IO-L3-2	.	.	.	.	.	.	.	.	.	.	.	.	.	A	A	.	.	.	C	.	A	.	-	-	-	-	-	-	T	-	-	-	-	-	-	-	-
BET-IO-L4O-2	-	-	-	-	-	-	-	-	-	-	-	-	-	-	-	-	-	-	-	-	-	-	-	-	.	.	.	.	T	.	.	.	C	.	.	C	C
BET-IO-L4B-2	-	-	-	-	-	-	-	-	-	-	-	-	-	-	-	-	-	-	-	-	-	-	-	-	.	.	.	.	T	.	.	.	C	.	.	C	C
BET-IO-L1-3	.	.	.	.	.	.	.	.	.	.	T	.	.	A	A	.	.	.	C	.	.	.	.	.	.	.	.	.	T	.	.	.	C	.	.	C	C
BET-IO-L2-3	.	.	.	.	.	.	.	.	.	.	T	.	.	A	A	.	.	.	C	.	.	.	.	.	.	.	.	.	T	.	.	.	C	.	.	C	C
BET-IO-L3-3	.	.	.	.	.	.	.	.	.	.	.	.	.	A	A	.	.	.	C	.	A	.	-	-	-	-	-	-	T	-	-	-	-	-	-	-	-
BET-IO-L4O-3	-	-	-	-	-	-	-	-	-	-	-	-	-	-	-	-	-	-	-	-	-	-	-	-	.	.	.	.	T	.	.	.	C	.	.	C	C
BET-IO-L4B-3	-	-	-	-	-	-	-	-	-	-	-	-	-	-	-	-	-	-	-	-	-	-	-	-	.	.	.	.	T	.	.	.	C	.	.	C	C
BET-EPO-L1-1	.	.	.	.	.	.	.	.	.	.	.	.	.	A	A	.	.	.	C	.	.	.	.	.	.	.	.	.	T	.	.	.	C	.	.	C	C
BET-EPO-L2-1	.	.	.	.	.	.	.	.	.	.	.	.	.	A	A	.	.	.	C	.	.	.	.	.	.	.	.	.	T	.	.	.	C	.	.	C	C
BET-EPO-L3-1	.	.	.	.	.	.	.	.	.	.	.	.	.	A	A	.	.	.	C	.	A	A	-	-	-	-	-	-	T	-	-	-	-	-	-	-	-
BET-EPO-L4O-1	-	-	-	-	-	-	-	-	-	-	-	-	-	-	-	-	-	-	-	-	-	-	-	-	.	.	.	.	T	.	C	.	C	.	.	C	C
BET-EPO-L4B-1	-	-	-	-	-	-	-	-	-	-	-	-	-	-	-	-	-	-	-	-	-	-	-	-	.	.	.	.	T	.	.	.	C	.	.	C	C
BET-EPO-L1-2	.	.	.	.	.	.	.	.	.	.	.	.	.	A	A	.	.	.	C	.	.	.	.	.	.	.	.	.	T	.	.	.	C	.	.	C	C
BET-EPO-L2-2	.	.	.	.	.	.	.	.	.	.	.	.	.	A	A	.	.	.	C	.	.	.	.	.	.	.	.	.	T	.	.	.	C	.	.	C	C
BET-EPO-L3-2	.	.	.	.	.	.	.	.	.	.	.	.	.	A	A	.	.	.	C	.	A	A	-	-	-	-	-	-	T	-	-	-	-	-	-	-	-
BET-EPO-L4O-2	-	-	-	-	-	-	-	-	-	-	-	-	-	-	-	-	-	-	-	-	-	-	-	-	.	.	.	.	T	.	.	.	C	.	.	C	C
BET-EPO-L4B-2	-	-	-	-	-	-	-	-	-	-	-	-	-	-	-	-	-	-	-	-	-	-	-	-	.	.	.	.	T	.	C	.	C	.	.	C	C
BET-EPO-L1-3	.	.	.	.	.	.	.	.	.	.	.	.	.	A	A	.	.	.	C	.	.	.	.	.	.	.	.	.	T	.	.	.	C	.	.	C	C
BET-EPO-L2-3	.	.	.	.	.	.	.	.	.	.	.	.	.	A	A	.	.	.	C	.	.	.	.	.	.	.	.	.	T	.	.	.	C	.	.	C	C
BET-EPO-L3-3	.	.	.	.	.	.	.	.	.	.	.	.	.	A	A	.	.	.	C	T	A	A	-	-	-	-	-	-	T	-	-	-	-	-	-	-	-
BET-EPO-L4O-3	-	-	-	-	-	-	-	-	-	-	-	-	-	-	-	-	-	-	-	-	-	-	-	-	.	.	.	.	T	.	.	.	C	.	.	C	C
BET-EPO-L4B-3	-	-	-	-	-	-	-	-	-	-	-	-	-	-	-	-	-	-	-	-	-	-	-	-	.	A	.	C	T	A	C	A	C	C	G	C	C

Supplementary Material

	2	6	9	18	21	30	33	36	39	45	63	69	72	75	84	90	93	96	102	105	108	109	111	120	123	129	132	144	151	156	162	174	180	183	192	198	213	219	231
NC 004901 T.thynnus	T	C	C	A	T	C	T	C	A	A	A	A	C	G	T	G	C	C	T	C	C	G	C	A	A	G	T	T	A	T	C	C	T	C	T	T	A	T	T
SKJ-AO-L1-1	C	T	T	T	C	T	C	.	C	.	T	C	A	T	A	.	T	T	C	T	.	.	A	.	.	.	A	.	.	C	T	A	C	A	C	C	G	C	C
SKJ-AO-L2-1	C	T	T	T	C	T	C	.	C	.	T	C	A	T	A	.	T	T	C	T	.	.	A	.	.	.	A	.	.	C	T	A	C	A	C	C	G	C	C
SKJ-AO-L3-1	C	T	T	T	C	T	C	.	C	.	T	C	A	T	A	.	T	T	C	T	.	.	A	.	.	.	A	.	.	C	T	A	C	A	C	C	G	C	C
SKJ-AO-L4O-1	C	T	T	T	C	T	C	.	C	.	T	C	A	T	A	A	T	T	C	.	.	.	A	-	-	-	-	-	-	-	-	-	-	-	-	-	-	-	-
SKJ-AO-L4B-1	-	-	-	-	-	-	-	-	-	-	-	-	-	-	-	-	-	-	-	-	-	-	-	-	-	.	.	.	G	.	T	.	.	.	C	.	.	C	.
SKJ-AO-L1-2	C	T	T	T	C	T	C	.	C	.	T	C	A	T	A	.	T	T	C	.	.	.	A	.	.	.	A	.	.	C	T	A	C	A	C	C	.	C	C
SKJ-AO-L2-2	C	T	T	T	C	T	C	.	C	.	T	C	A	T	A	.	T	T	C	.	.	.	A	.	.	.	A	.	.	C	T	A	C	A	C	C	.	C	C
SKJ-AO-L3-2	C	T	T	T	C	T	C	.	C	.	T	C	A	T	A	.	T	T	C	.	.	.	A	.	.	.	A	.	.	C	T	A	C	A	C	C	.	C	C
SKJ-AO-L4O-2	-	-	-	-	-	-	-	-	-	-	-	-	-	-	-	-	-	-	-	-	-	-	-	-	-	.	.	.	.	C	T	A	.	.	C	C	.	C	C
SKJ-AO-L4B-2	-	-	-	-	-	-	-	-	-	-	-	-	-	-	-	-	-	-	-	-	-	-	-	-	-	.	.	.	.	.	T	.	.	.	C	.	.	C	C
SKJ-AO-L1-3	C	T	T	T	C	T	C	.	C	.	T	C	A	C	A	.	T	T	C	T	.	.	A	.	.	.	A	.	.	C	T	A	C	A	C	C	G	C	C
SKJ-AO-L2-3	C	T	T	T	C	T	C	.	C	.	T	C	A	C	A	.	T	T	C	T	.	.	A	.	.	.	A	.	.	C	T	A	C	A	C	C	G	C	C
SKJ-AO-L3-3	-	-	-	-	-	-	-	-	-	-	-	-	-	-	-	-	-	-	-	-	-	-	-	-	-	-	-	-	-	-	-	-	-	-	-	-	-	-	-
SKJ-AO-L4O-3	-	-	-	-	-	-	-	-	-	-	-	-	-	-	-	-	-	-	-	-	-	-	-	-	-	.	A	.	.	C	T	A	C	A	C	C	G	C	C
SKJ-AO-L4B-3	-	-	-	-	-	-	-	-	-	-	-	-	-	-	-	-	-	-	-	-	-	-	-	-	-	.	A	.	.	C	T	A	C	A	C	C	.	C	C
SKJ-IO-L1-1	C	T	T	T	C	T	C	.	C	.	T	C	A	T	A	.	T	T	C	.	.	.	A	.	G	.	A	.	.	C	T	A	C	A	C	C	G	C	C
SKJ-IO-L2-1	C	T	T	T	C	T	C	.	C	.	T	C	A	T	A	.	T	T	C	.	.	.	A	.	G	.	A	.	.	C	T	A	C	A	C	C	G	C	C
SKJ-IO-L3-1	C	T	T	T	C	T	C	.	C	.	T	C	A	T	A	.	T	T	C	.	.	.	A	.	G	.	A	.	.	C	T	A	C	A	C	C	G	C	C
SKJ-IO-L4O-1	-	-	-	-	-	-	-	-	-	-	-	-	-	-	-	-	-	-	-	-	-	-	-	-	-	.	.	.	G	.	T	.	.	.	C	.	.	C	C
SKJ-IO-L4B-1	-	-	-	-	-	-	-	-	-	-	-	-	-	-	-	-	-	-	-	-	-	-	-	-	-	.	A	.	.	C	T	.	.	A	C	.	.	C	C
SKJ-IO-L1-2	C	T	T	T	C	T	C	.	C	.	T	C	A	T	A	.	T	T	C	T	.	.	A	.	.	.	A	.	.	C	T	A	C	A	C	C	G	C	C
SKJ-IO-L2-2	C	T	T	T	C	T	C	.	C	.	T	C	A	T	A	.	T	T	C	T	.	.	A	.	.	.	A	.	.	C	T	A	C	A	C	C	G	C	C
SKJ-IO-L3-2	C	T	T	T	C	T	C	.	C	.	T	C	A	T	A	.	T	T	C	T	.	.	A	.	.	.	A	.	.	C	T	A	C	A	C	C	G	C	C
SKJ-IO-L4O-2	-	-	-	-	-	-	-	-	-	-	-	-	-	-	-	-	-	-	-	-	-	-	-	-	-	.	.	.	.	.	T	.	.	.	C	.	.	C	C
SKJ-IO-L4B-2	-	-	-	-	-	-	-	-	-	-	-	-	-	-	-	-	-	-	-	-	-	-	-	-	-	-	-	-	-	-	-	-	-	-	-	-	-	-	-
SKJ-IO-L1-3	C	T	T	T	C	T	C	.	C	.	T	C	A	T	A	.	T	T	C	.	.	.	A	.	G	.	A	.	.	C	T	A	C	A	C	C	G	C	C
SKJ-IO-L2-3	C	T	T	T	C	T	C	.	C	.	T	C	A	T	A	.	T	T	C	.	.	.	A	.	G	.	A	.	.	C	T	A	C	A	C	C	G	C	C
SKJ-IO-L3-3	C	T	T	T	C	T	C	.	C	.	T	C	A	T	A	.	T	T	C	.	.	.	A	.	G	.	A	.	.	C	T	A	C	A	C	C	G	C	C
SKJ-IO-L4O-3	-	-	-	-	-	-	-	-	-	-	-	-	-	-	-	-	-	-	-	-	-	-	-	-	-	.	A	.	.	C	T	A	C	A	C	C	G	C	C
SKJ-IO-L4B-3	-	-	-	-	-	-	-	-	-	-	-	-	-	-	-	-	-	-	-	-	-	-	-	-	-	.	A	.	.	C	T	A	C	A	C	C	G	C	C
SKJ-WCPO-L1-1	C	T	T	T	C	T	C	.	C	.	T	C	A	T	A	.	T	T	C	T	.	.	A	.	.	.	A	.	.	C	T	A	C	A	C	C	G	C	C
SKJ-WCPO-L2-1	C	T	T	T	C	T	C	.	C	.	T	C	A	T	A	.	T	T	C	T	.	.	A	.	.	.	A	.	.	C	T	A	C	A	C	C	G	C	C
SKJ-WCPO-L3-1	C	T	T	T	C	T	C	.	C	.	T	C	A	T	A	.	T	T	C	.	.	.	A	.	.	.	A	.	.	C	T	A	C	A	C	C	.	C	C
SKJ-WCPO-L4O-1	-	-	-	-	-	-	-	-	-	-	-	-	-	-	-	-	-	-	-	-	-	-	-	-	-	.	.	.	.	.	T	.	.	.	C	.	.	C	C
SKJ-WCPO-L4B-1	-	-	-	-	-	-	-	-	-	-	-	-	-	-	-	-	-	-	-	-	-	-	-	-	-	.	.	.	.	.	T	.	.	.	C	.	.	C	C
SKJ-WCPO-L1-2	C	T	T	T	C	T	C	.	C	.	T	C	A	T	A	.	T	T	C	.	.	.	A	.	.	.	A	.	.	C	T	A	C	A	C	C	.	C	C
SKJ-WCPO-L2-2	C	T	T	T	C	T	C	.	C	.	T	C	A	T	A	.	T	T	C	.	.	.	A	.	.	.	A	.	.	C	T	A	C	A	C	C	.	C	C
SKJ-WCPO-L3-2	C	T	T	T	C	T	C	.	C	.	T	C	A	T	A	A	T	T	C	.	.	.	A	.	.	.	A	.	.	C	T	A	C	A	C	C	.	C	C
SKJ-WCPO-L4O-2	-	-	-	-	-	-	-	-	-	-	-	-	-	-	-	-	-	-	-	-	-	-	-	-	-	.	A	.	.	C	T	A	C	A	C	C	.	C	C
SKJ-WCPO-L4B-2	-	-	-	-	-	-	-	-	-	-	-	-	-	-	-	-	-	-	-	-	-	-	-	-	-	.	.	.	.	.	T	.	.	.	C	.	.	C	C

Supplementary Material

	2	6	9	18	21	30	33	36	39	45	63	69	72	75	84	90	93	96	102	105	108	109	111	120	123	129	132	144	151	156	162	174	180	183	192	198	213	219	231
NC 004901 T.thynnus	T	C	C	A	T	C	T	C	A	A	A	A	C	G	T	G	C	C	T	C	C	G	C	A	A	G	T	T	A	T	C	C	T	C	T	T	A	T	T
SKJ-WCPO-L1-3	C	T	T	T	C	T	C	.	C	.	T	C	A	T	A	.	T	T	C	.	.	.	A	.	G	.	A	.	.	C	T	A	C	A	C	C	.	C	C
SKJ-WCPO-L2-3	C	T	T	T	C	T	C	.	C	.	T	C	A	T	A	.	T	T	C	.	.	.	A	.	G	.	A	.	.	C	T	A	C	A	C	C	.	C	C
SKJ-WCPO-L3-3	C	T	T	T	C	T	C	.	C	.	T	T	A	T	A	.	T	T	C	.	T	.	A	.	G	.	A	.	.	C	T	A	C	A	C	C	.	C	C
SKJ-WCPO-L4O-3	-	-	-	-	-	-	-	-	-	-	-	-	-	-	-	-	-	-	-	-	-	-	-	-	-	.	A	.	.	C	T	A	C	A	C	C	.	C	C
SKJ-WCPO-L4B-3	-	-	-	-	-	-	-	-	-	-	-	-	-	-	-	-	-	-	-	-	-	-	-	-	-	.	C	.	.	C	T	A	C	A	C	C	.	C	C
SKJ-EPO-L1-1	C	T	T	T	C	T	C	.	C	.	T	C	A	T	A	.	T	T	C	T	.	.	A	.	.	.	A	.	.	C	T	A	C	A	C	C	.	C	C
SKJ-EPO-L2-1	C	T	T	T	C	T	C	.	C	.	T	C	A	T	A	.	T	T	C	T	.	.	A	.	.	.	A	.	.	C	T	A	C	A	C	C	.	C	C
SKJ-EPO-L3-1	C	T	T	T	C	T	C	.	C	.	T	C	A	T	A	.	T	T	C	T	.	.	A	.	.	.	A	.	.	C	T	A	C	A	C	C	G	C	C
SKJ-EPO-L4O-1	-	-	-	-	-	-	-	-	-	-	-	-	-	-	-	-	-	-	-	-	-	-	-	-	-	.	.	.	.	.	T	.	.	.	C	.	.	C	C
SKJ-EPO-L4B-1	-	-	-	-	-	-	-	-	-	-	-	-	-	-	-	-	-	-	-	-	-	-	-	-	-	.	.	.	.	.	T	.	.	.	C	.	.	C	C
SKJ-EPO-L1-2	C	T	T	T	C	T	C	.	C	.	T	C	A	T	A	A	T	T	C	.	.	C	A	.	.	.	A	.	.	C	T	A	C	A	C	C	.	C	C
SKJ-EPO-L2-2	C	T	T	T	C	T	C	.	C	.	T	C	A	T	A	A	T	T	C	.	.	.	A	.	.	.	A	.	.	C	T	A	C	A	C	C	.	C	C
SKJ-EPO-L3-2	C	T	T	T	C	T	C	.	C	.	T	C	A	T	A	A	T	T	C	.	.	.	.	.	.	.	.	.	.	.	T	.	.	.	C	.	.	C	C
SKJ-EPO-L4O-2	-	-	-	-	-	-	-	-	-	-	-	-	-	-	-	-	-	-	-	-	-	-	-	-	-	.	.	.	.	.	T	.	.	.	C	.	.	C	C
SKJ-EPO-L4B-2	-	-	-	-	-	-	-	-	-	-	-	-	-	-	-	-	-	-	-	-	-	-	-	-	-	.	.	.	.	.	T	.	.	.	C	.	.	C	C
SKJ-EPO-L1-3	C	T	T	T	C	T	C	.	C	.	T	C	A	T	A	.	T	T	C	.	T	.	A	.	G	.	A	.	.	C	T	A	C	A	C	C	.	C	C
SKJ-EPO-L2-3	C	T	T	T	C	T	C	.	C	.	T	C	A	T	A	.	T	T	C	.	T	.	A	.	G	.	A	.	.	C	T	A	C	A	C	C	.	C	C
SKJ-EPO-L3-3	C	T	T	T	C	T	C	.	C	.	T	C	A	T	A	.	T	T	C	T	.	.	A	.	.	.	A	.	.	C	T	A	C	A	C	C	.	C	C
SKJ-EPO-L4O-3	-	-	-	-	-	-	-	-	-	-	-	-	-	-	-	-	-	-	-	-	-	-	-	-	-	.	A	.	.	C	T	A	C	A	C	C	.	C	C
SKJ-EPO-L4B-3	-	-	-	-	-	-	-	-	-	-	-	-	-	-	-	-	-	-	-	-	-	-	-	-	-	.	A	.	.	C	T	A	C	A	C	C	.	C	C

Supplementary Material

	2	6	9	18	21	30	33	36	39	42	45	63	69	72	75	84	93	96	102	105	111
NC 004901 T.thynnus	T	C	C	A	T	C	T	C	A	C	A	A	A	C	G	T	C	C	T	C	C
LOT-IO-L1-1	.	.	.	.	.	.	.	.	.	.	.	.	.	.	T	.	.	.	.	.	.
LOT-IO-L2-1	.	.	.	.	.	.	.	.	.	.	.	.	.	.	T	.	.	.	.	.	.
LOT-IO-L3-1	.	.	.	.	.	.	.	.	.	.	.	.	.	.	T	.	.	.	C	.	A
LOT-IO-L4O-1	-	-	-	-	-	-	-	-	-	-	-	-	-	-	-	-	-	-	-	-	-
LOT-IO-L4B-1	-	-	-	-	-	-	-	-	-	-	-	-	-	-	-	-	-	-	-	-	-
LOT-IO-L1-2	.	.	.	.	.	.	.	.	.	.	.	.	.	.	T	.	.	.	.	.	.
LOT-IO-L2-2	.	.	.	.	.	.	.	.	.	.	.	.	.	.	T	.	.	.	.	.	.
LOT-IO-L3-2	.	.	.	.	.	.	.	.	.	T	.	.	.	.	T	.	.	.	.	.	.
LOT-IO-L4O-2	-	-	-	-	-	-	-	-	-	-	-	-	-	-	-	-	-	-	-	-	-
LOT-IO-L4B-2	-	-	-	-	-	-	-	-	-	-	-	-	-	-	-	-	-	-	-	-	-
LOT-IO-L1-3	.	.	.	.	.	T	.	.	.	.	.	.	.	.	T	.	.	.	.	.	.
LOT-IO-L2-3	.	.	.	.	.	.	.	.	.	T	.	.	.	.	T	.	.	.	.	.	.
LOT-IO-L3-3	.	.	.	.	.	.	.	.	.	T	.	.	.	.	T	.	.	.	.	.	.
LOT-IO-L4O-3	-	-	-	-	-	-	-	-	-	-	-	-	-	-	-	-	-	-	-	-	-
LOT-IO-L4B-3	-	-	-	-	-	-	-	-	-	-	-	-	-	-	-	-	-	-	-	-	-

	120	123	129	132	144	156	162	174	180	183	192	198	213	219	231
NC 004901 T.thynnus	A	A	G	T	T	T	C	C	T	C	T	T	A	T	T
LOT-IO-L1-1	G	.	.	.	.	.	T	.	.	.	.	.	.	C	C
LOT-IO-L2-1	G	.	.	.	.	.	T	.	.	.	.	.	.	C	C
LOT-IO-L3-1	-	-	-	-	-	-	-	-	-	-	-	-	-	-	-
LOT-IO-L4O-1	-	-	.	.	.	.	T	.	.	.	C	.	.	C	C
LOT-IO-L4B-1	-	-	.	.	.	.	T	.	.	.	C	.	.	C	C
LOT-IO-L1-2	G	.	.	.	.	.	T	.	.	.	.	.	.	C	C
LOT-IO-L2-2	G	.	.	.	.	.	T	.	.	.	.	.	.	C	C
LOT-IO-L3-2	.	.	.	.	.	.	T	.	.	.	.	.	.	.	C
LOT-IO-L4O-2	-	-	.	.	.	.	T	.	.	.	.	.	.	.	C
LOT-IO-L4B-2	-	-	.	.	.	.	T	.	.	.	C	.	.	.	C
LOT-IO-L1-3	G	.	.	.	.	.	T	.	.	.	.	.	.	C	C
LOT-IO-L2-3	.	.	.	.	.	.	T	.	.	.	.	.	.	C	C
LOT-IO-L3-3	.	.	.	.	.	.	T	.	.	.	.	.	.	C	C
LOT-IO-L4O-3	-	-	.	.	.	.	T	.	.	.	.	.	.	.	C
LOT-IO-L4B-3	-	-	.	.	.	.	T	.	.	.	.	.	.	.	C
