# Supplementary material for: Canning Processes Reduce the DNA-Based Traceability of Commercial Tropical Tunas
Source: Foods. 2020 Sep 27;9(10):1372. doi: 10.3390/foods9101372 (PMC7650566; doi:10.3390/foods9101372)
Supplement: Supplementary file 1 [file foods-09-01372-s001.zip › SUPPLEMENTARY/Table S3_foods-943909_rev18_09_2020.pdf]

Pecoraro et al. Canning processes reduce DNA-based traceability of commercial tropical tunas

Table S3: Summary information of the samples used in this study and their molecular identification results. Morph. ID = Morphological identification for the four species (*Thunnus albacares* = YFT; *Thunnus obesus* = BET; *Katsuwonus pelamis* = SKJ; *Thunnus tonggol* = LOT;) achieved at the canneries; Ocean (Indian Ocean = IO; Atlantic Ocean = AO; Western-Central Pacific Ocean = WCPO; Eastern Pacific Ocean = EPO); amplified fragment of the of the mitochondrial gene Cytochrome b (AB, A, B); Blast ID = species genetically identified in GenBank (www.ncbi.nlm.nih.gov) using the BLASTn search tool; % Similarity = genetic similarity in GenBank; Accession number of the species genetically identified in GenBank.

Morph. ID	Ocean	Level	Scpecimen	Fragment	Blast ID	% Similarity	Accession number
**YFT**	**AO**	**L1**	1	AB	*Thunnus albacares*	99.58%	MG017682.1
**YFT**	**AO**	**L2**	1	AB	*Thunnus albacares*	99.58%	MG017682.1
**YFT**	**AO**	**L3**	1	AB	*Thunnus albacares*	99.15%	MG017682.1
**YFT**	**AO**	**L4B**	1	B	*Katsuwonus pelamis*	100.00%	KP669132.1
**YFT**	**AO**	**L4O**	1	B	*Thunnus obesus*	98.17%	MG017696.1
**YFT**	**AO**	**L1**	2	AB	*Thunnus albacares*	99.58%	MG017682.1
**YFT**	**AO**	**L2**	2	AB	*Thunnus albacares*	99.58%	MG017682.1
**YFT**	**AO**	**L3**	2	AB	*Thunnus albacares*	99.58%	MG017682.1
**YFT**	**AO**	**L4B**	2	B	*Katsuwonus pelamis*	100.00%	KP669132.1
**YFT**	**AO**	**L4O**	2	B	*Katsuwonus pelamis*	100.00%	KP669132.1
**YFT**	**AO**	**L1**	3	AB	*Thunnus albacares*	98.31%	KX755269.1
**YFT**	**AO**	**L2**	3	AB	*Thunnus albacares*	98.31%	MG017682.1
**YFT**	**AO**	**L3**	3	-	-	-	-
**YFT**	**AO**	**L4B**	3	B	*Katsuwonus pelamis*	100.00%	KP669132.1
**YFT**	**AO**	**L4O**	3	B	*Thunnus albacares*	99.08%	MG017686.1
**YFT**	**EPO**	**L1**	1	AB	*Thunnus albacares*	99.15%	MG017682.1
**YFT**	**EPO**	**L2**	1	AB	*Thunnus albacares*	99.15%	MG017682.1
**YFT**	**EPO**	**L3**	1	AB	*Thunnus albacares*	99.15%	MG017682.1
**YFT**	**EPO**	**L4B**	1	B	*Katsuwonus pelamis, Thunnus albacares, Thunnus obesus*	96.33%	KP669132.1, MG017683.1, KJ018958.1
**YFT**	**EPO**	**L4O**	1	B	*Thunnus albacares*	98.17%	MG017683.1
**YFT**	**EPO**	**L1**	2	AB	*Thunnus albacares*	99.15%	MG017682.1
**YFT**	**EPO**	**L2**	2	AB	*Thunnus albacares*	99.15%	MG017682.1
**YFT**	**EPO**	**L3**	2	AB	*Thunnus albacares*	99.15%	MG017682.1
**YFT**	**EPO**	**L4B**	2	B	*Katsuwonus pelamis*	99.08%	KP669132.1
**YFT**	**EPO**	**L4O**	2	B	*Thunnus albacares*	98.17%	MG017683.1
**YFT**	**EPO**	**L1**	3	AB	*Thunnus albacares*	99.15%	MG017682.1
**YFT**	**EPO**	**L2**	3	AB	*Thunnus albacares*	99.15%	MG017682.1
**YFT**	**EPO**	**L3**	3	AB	*Thunnus albacares*	99.15%	MG017682.1
**YFT**	**EPO**	**L4B**	3	B	*Thunnus albacares*	97.25%	MG017683.1
**YFT**	**EPO**	**L4O**	3	B	*Thunnus albacares*	98.17%	MG017683.1
**YFT**	**IO**	**L1**	1	AB	*Thunnus albacares*	99.58%	MG017682.1
**YFT**	**IO**	**L2**	1	AB	*Thunnus albacares*	99.58%	MG017682.1
**YFT**	**IO**	**L3**	1	AB	*Thunnus albacares*	99.58%	MG017682.1
**YFT**	**IO**	**L4B**	1	B	*Thunnus albacares*	99.08%	MG017683.1
**YFT**	**IO**	**L4O**	1	B	*Katsuwonus pelamis*	98.17%	KP669172.1
**YFT**	**IO**	**L1**	2	AB	*Thunnus albacares*	99.58%	MG017682.1
**YFT**	**IO**	**L2**	2	AB	*Thunnus albacares*	99.58%	MG017682.1
**YFT**	**IO**	**L3**	2	AB	*Thunnus albacares*	99.58%	MG017682.1
**YFT**	**IO**	**L4B**	2	B	*Thunnus albacares, T.obesus*	97.25%	MG017683.1, MG017696.1
**YFT**	**IO**	**L4O**	2	B	*Thunnus albacares*	99.08%	MG017683.1
**YFT**	**IO**	**L1**	3	AB	*Thunnus albacares*	99.58%	MG017682.1
**YFT**	**IO**	**L2**	3	AB	*Thunnus albacares*	99.58%	MG017682.1
**YFT**	**IO**	**L3**	3	AB	*Thunnus albacares*	99.58%	MG017682.1
**YFT**	**IO**	**L4B**	3	B	*Katsuwonus pelamis*	99.08%	KP669172.1
**YFT**	**IO**	**L4O**	3	B	*Thunnus albacares*	99.08%	MG017686.1

Supplementary Material

Morph. ID	Ocean	Level	Scpecimen	Fragment	Blast ID	% Similarity	Accession number
**YFT**	**WCPO**	**L1**	1	AB	*Thunnus albacares*	99.58%	MG017682.1
**YFT**	**WCPO**	**L2**	1	AB	*Thunnus albacares*	99.58%	MG017682.1
**YFT**	**WCPO**	**L3**	1	AB	*Thunnus albacares*	99.58%	MG017682.1
**YFT**	**WCPO**	**L4B**	1	B	*Katsuwonus pelamis*	99.08%	KP669132.1
**YFT**	**WCPO**	**L4O**	1	B	*Thunnus albacares*	99.08%	MG017683.1
**YFT**	**WCPO**	**L1**	2	AB	*Thunnus albacares*	99.58%	MG017682.1
**YFT**	**WCPO**	**L2**	2	AB	*Thunnus albacares*	99.58%	MG017682.1
**YFT**	**WCPO**	**L3**	2	AB	*Thunnus albacares*	99.58%	MG017682.1
**YFT**	**WCPO**	**L4B**	2	B	*Thunnus albacares*	99.08%	MG017683.1
**YFT**	**WCPO**	**L4O**	2	B	*Thunnus albacares*	99.08%	MG017683.1
**YFT**	**WCPO**	**L1**	3	AB	*Thunnus albacares*	99.58%	MG017682.1
**YFT**	**WCPO**	**L2**	3	AB	*Thunnus albacares*	99.58%	MG017682.1
**YFT**	**WCPO**	**L3**	3	AB	*Thunnus albacares*	99.58%	MG017682.1
**YFT**	**WCPO**	**L4B**	3	B	*Thunnus albacares, T.obesus*	97.25%	MG017683.1, KJ018958.1
**YFT**	**WCPO**	**L4O**	3	B	*Thunnus albacares*	99.08%	MG017686.1
**BET**	**EPO**	**L1**	1	AB	*Thunnus obesus*	99.15%	MG017695.1
**BET**	**EPO**	**L2**	1	AB	*Thunnus obesus*	99.15%	MG017695.1
**BET**	**EPO**	**L3**	1	A	*Thunnus obesus*	99.14%	MG017697.1
**BET**	**EPO**	**L4B**	1	B	*Thunnus obesus*	98.17%	MG017696.1
**BET**	**EPO**	**L4O**	1	B	*Thunnus obesus*	98.17%	MG017697.1
**BET**	**EPO**	**L1**	2	AB	*Thunnus obesus*	99.15%	MG017695.1
**BET**	**EPO**	**L2**	2	AB	*Thunnus obesus*	99.15%	MG017695.1
**BET**	**EPO**	**L3**	2	A	*Thunnus obesus*	99.14%	MG017697.1
**BET**	**EPO**	**L4B**	2	B	*Thunnus obesus*	98.17%	MG017697.1
**BET**	**EPO**	**L4O**	2	B	*Thunnus obesus*	98.17%	MG017696.1
**BET**	**EPO**	**L1**	3	AB	*Thunnus obesus*	99.15%	MG017695.1
**BET**	**EPO**	**L2**	3	AB	*Thunnus obesus*	99.15%	MG017695.1
**BET**	**EPO**	**L3**	3	A	*Thunnus obesus*	98.28%	MG017697.1
**BET**	**EPO**	**L4B**	3	B	*Katsuwonus pelamis*	100.00%	AB098093.1
**BET**	**EPO**	**L4O**	3	B	*Thunnus obesus*	98.17%	MG017696.1
**BET**	**IO**	**L1**	1	AB	*Thunnus obesus*	99.15%	MG017695.1
**BET**	**IO**	**L2**	1	AB	*Thunnus obesus*	99.15%	MG017695.1
**BET**	**IO**	**L3**	1	A	*Thunnus obesus*	99.15%	MG017697.1
**BET**	**IO**	**L4B**	1	B	*Thunnus obesus*	98.17%	MG017696.1
**BET**	**IO**	**L4O**	1	B	*Thunnus obesus*	98.17%	MG017696.1
**BET**	**IO**	**L1**	2	AB	*Thunnus obesus*	99.15%	MG017695.1
**BET**	**IO**	**L2**	2	AB	*Thunnus obesus*	99.15%	MG017697.1
**BET**	**IO**	**L3**	2	A	*Thunnus obesus*	99.15%	MG017697.1
**BET**	**IO**	**L4B**	2	B	*Thunnus obesus*	98.17%	MG017696.1
**BET**	**IO**	**L4O**	2	B	*Thunnus obesus*	98.17%	MG017696.1
**BET**	**IO**	**L1**	3	AB	*Thunnus obesus*	98.73%	MG017695.1
**BET**	**IO**	**L2**	3	AB	*Thunnus obesus*	98.73%	MG017695.1
**BET**	**IO**	**L3**	3	A	*Thunnus obesus*	99.15%	MG017697.1
**BET**	**IO**	**L4B**	3	B	*Thunnus obesus*	98.17%	MG017696.1
**BET**	**IO**	**L4O**	3	B	*Thunnus obesus*	98.17%	MG017696.1
**SKJ**	**AO**	**L1**	1	AB	*Katsuwonus pelamis*	99.58%	KP669170.1
**SKJ**	**AO**	**L2**	1	AB	*Katsuwonus pelamis*	99.58%	KP669170.1
**SKJ**	**AO**	**L3**	1	AB	*Katsuwonus pelamis*	99.58%	KP669170.1
**SKJ**	**AO**	**L4B**	1	B	*Thunnus obesus*	98.17%	MG017696.1
**SKJ**	**AO**	**L4O**	1	A	*Katsuwonus pelamis*	100.00%	KJ617313.1
**SKJ**	**AO**	**L1**	2	AB	*Katsuwonus pelamis*	99.58%	KP669130.1
**SKJ**	**AO**	**L2**	2	AB	*Katsuwonus pelamis*	99.15%	KP669130.1
**SKJ**	**AO**	**L3**	2	AB	*Katsuwonus pelamis*	99.58%	KP669130.1
**SKJ**	**AO**	**L4B**	2	B	*Thunnus obesus*	98.17%	MG017696.1
**SKJ**	**AO**	**L4O**	2	B	*Katsuwonus pelamis*	97.25%	KP669132.1

Supplementary Material

Morph. ID	Ocean	Level	Scpecimen	Fragment	Blast ID	% Similarity	Accession number
**SKJ**	**AO**	**L1**	3	AB	*Katsuwonus pelamis*	99.58%	KP669170.1
**SKJ**	**AO**	**L2**	3	AB	*Katsuwonus pelamis*	99.58%	KP669170.1
**SKJ**	**AO**	**L3**	3	-	-	-	-
**SKJ**	**AO**	**L4B**	3	B	*Katsuwonus pelamis*	100.00%	KP669132.1
**SKJ**	**AO**	**L4O**	3	B	*Katsuwonus pelamis*	100.00%	AB098093.1
**SKJ**	**EPO**	**L1**	1	AB	*Katsuwonus pelamis*	100.00%	KJ617298.1
**SKJ**	**EPO**	**L2**	1	AB	*Katsuwonus pelamis*	100.00%	KJ617298.1
**SKJ**	**EPO**	**L3**	1	AB	*Katsuwonus pelamis*	99.58%	KP669170.1
**SKJ**	**EPO**	**L4B**	1	B	*Thunnus obesus*	98.17%	MG017696.1
**SKJ**	**EPO**	**L4O**	1	B	*Thunnus obesus*	98.17%	MG017696.1
**SKJ**	**EPO**	**L1**	2	AB	*Katsuwonus pelamis*	98.31%	KP669130.1
**SKJ**	**EPO**	**L2**	2	AB	*Katsuwonus pelamis*	98.31%	KP669130.1
**SKJ**	**EPO**	**L3**	2	AB	*Katsuwonus pelamis*	96.19%	KP669130.1
**SKJ**	**EPO**	**L4B**	2	B	*Thunnus obesus*	98.17%	MG017696.1
**SKJ**	**EPO**	**L4O**	2	B	*Thunnus obesus*	98.17%	MG017696.1
**SKJ**	**EPO**	**L1**	3	AB	*Katsuwonus pelamis*	99.15%	KP669172.1
**SKJ**	**EPO**	**L2**	3	AB	*Katsuwonus pelamis*	99.15%	KP669172.1
**SKJ**	**EPO**	**L3**	3	AB	*Katsuwonus pelamis*	100.00%	KJ617298.1
**SKJ**	**EPO**	**L4B**	3	B	*Katsuwonus pelamis*	100.00%	KP669132.1
**SKJ**	**EPO**	**L4O**	3	B	*Katsuwonus pelamis*	100.00%	KP669132.1
**SKJ**	**IO**	**L1**	1	AB	*Katsuwonus pelamis*	100.00%	AB098093.1
**SKJ**	**IO**	**L2**	1	AB	*Katsuwonus pelamis*	100.00%	AB098093.1
**SKJ**	**IO**	**L3**	1	AB	*Katsuwonus pelamis*	100.00%	AB098093.1
**SKJ**	**IO**	**L4B**	1	B	*Katsuwonus pelamis*	97.25%	KP669132.1
**SKJ**	**IO**	**L4O**	1	B	*Thunnus obesus*	98.17%	MG017696.1
**SKJ**	**IO**	**L1**	2	AB	*Katsuwonus pelamis*	99.15%	KP669170.1
**SKJ**	**IO**	**L2**	2	AB	*Katsuwonus pelamis*	99.58%	KP669170.1
**SKJ**	**IO**	**L3**	2	AB	*Katsuwonus pelamis*	99.58%	KP669170.1
**SKJ**	**IO**	**L4B**	2	-	-	-	-
**SKJ**	**IO**	**L4O**	2	B	*Thunnus obesus*	98.17%	MG017696.1
**SKJ**	**IO**	**L1**	3	AB	*Katsuwonus pelamis*	100.00%	AB098093.1
**SKJ**	**IO**	**L2**	3	AB	*Katsuwonus pelamis*	100.00%	AB098093.1
**SKJ**	**IO**	**L3**	3	AB	*Katsuwonus pelamis*	100.00%	AB098093.1
**SKJ**	**IO**	**L4B**	3	B	*Katsuwonus pelamis*	100.00%	KP669132.1
**SKJ**	**IO**	**L4O**	3	B	*Katsuwonus pelamis*	100.00%	KP669132.1
**SKJ**	**WCPO**	**L1**	1	AB	*Katsuwonus pelamis*	99.58%	KP669170.1
**SKJ**	**WCPO**	**L2**	1	AB	*Katsuwonus pelamis*	99.58%	KP669170.1
**SKJ**	**WCPO**	**L3**	1	AB	*Katsuwonus pelamis*	99.58%	KP669130.1
**SKJ**	**WCPO**	**L4B**	1	B	*Thunnus obesus*	98.17%	MG017696.1
**SKJ**	**WCPO**	**L4O**	1	B	*Thunnus obesus*	98.17%	MG017696.1
**SKJ**	**WCPO**	**L1**	2	AB	*Katsuwonus pelamis*	99.15%	KP669130.1
**SKJ**	**WCPO**	**L2**	2	AB	*Katsuwonus pelamis*	99.58%	KP669130.1
**SKJ**	**WCPO**	**L3**	2	AB	*Katsuwonus pelamis*	99.58%	KP669130.1
**SKJ**	**WCPO**	**L4B**	2	B	*Thunnus obesus*	98.17%	MG017696.1
**SKJ**	**WCPO**	**L4O**	2	B	*Katsuwonus pelamis*	100.00%	KP669132.1
**SKJ**	**WCPO**	**L1**	3	AB	*Katsuwonus pelamis*	99.58%	KP669170.1
**SKJ**	**WCPO**	**L2**	3	AB	*Katsuwonus pelamis*	99.58%	KP669170.1
**SKJ**	**WCPO**	**L3**	3	AB	*Katsuwonus pelamis*	99.15%	AB098096.1
**SKJ**	**WCPO**	**L4B**	3	B	*Katsuwonus pelamis*	99.08%	KP669132.1
**SKJ**	**WCPO**	**L4O**	3	B	*Katsuwonus pelamis*	100.00%	KP669132.1

Supplementary Material

Morph. ID	Ocean	Level	Scpecimen	Fragment	Blast ID	% Similarity	Accession number
**LOT**	**IO**	**L1**	1	AB	*Thunnus tonggol, thunnus atlanticus*	98.73%	KM055411.1, MG017704.1
**LOT**	**IO**	**L2**	1	AB	*Thunnus tonggol, thunnus atlanticus*	98.73%	KM055411.1, MG017704.1
**LOT**	**IO**	**L3**	1	A	*Thunnus tonggol*	98.29%	KF777796.1
**LOT**	**IO**	**L4B**	1	B	*Thunnus obesus*	98.17%	MG017696.1
**LOT**	**IO**	**L4O**	1	B	*Thunnus obesus*	98.17%	MG017696.1
**LOT**	**IO**	**L1**	2	AB	*Thunnus tonggol*	98.73%	KM055411.1
**LOT**	**IO**	**L2**	2	AB	*Thunnus tonggol*	98.73%	KM055411.1
**LOT**	**IO**	**L3**	2	AB	*Thunnus thynnus, Thunnus albacares*	98.73%	MG017705.1, MG017687.1
**LOT**	**IO**	**L4B**	2	B	*Thunnus obesus*	98.17%	MG017696.1
**LOT**	**IO**	**L4O**	2	B	*Thunnus tonggol, Thunnus thynnus*	99.08%	MG017702.1, MG017705.1
**LOT**	**IO**	**L1**	3	AB	*Thunnus tonggol, thunnus atlanticus*	98.31%	KM055411.1, MG017704.1
**LOT**	**IO**	**L2**	3	AB	*Thunnus thynnus, Thunnus albacares*	98.73%	MG017705.1, MG017687.1
**LOT**	**IO**	**L3**	3	AB	*Thunnus thynnus, Thunnus albacares*	98.73%	MG017705.1, MG017687.1
**LOT**	**IO**	**L4B**	3	B	*Thunnus tonggol, Thunnus thynnus*	99.08%	MG017702.1, MG017705.1
**LOT**	**IO**	**L4O**	3	B	*Thunnus tonggol, Thunnus thynnus*	99.08%	MG017702.1, MG017705.1
