# Supplementary figures and images for: Canning Processes Reduce the DNA-Based Traceability of Commercial Tropical Tunas
Source: Foods. 2020 Sep 27;9(10):1372. doi: 10.3390/foods9101372 (PMC7650566; doi:10.3390/foods9101372)

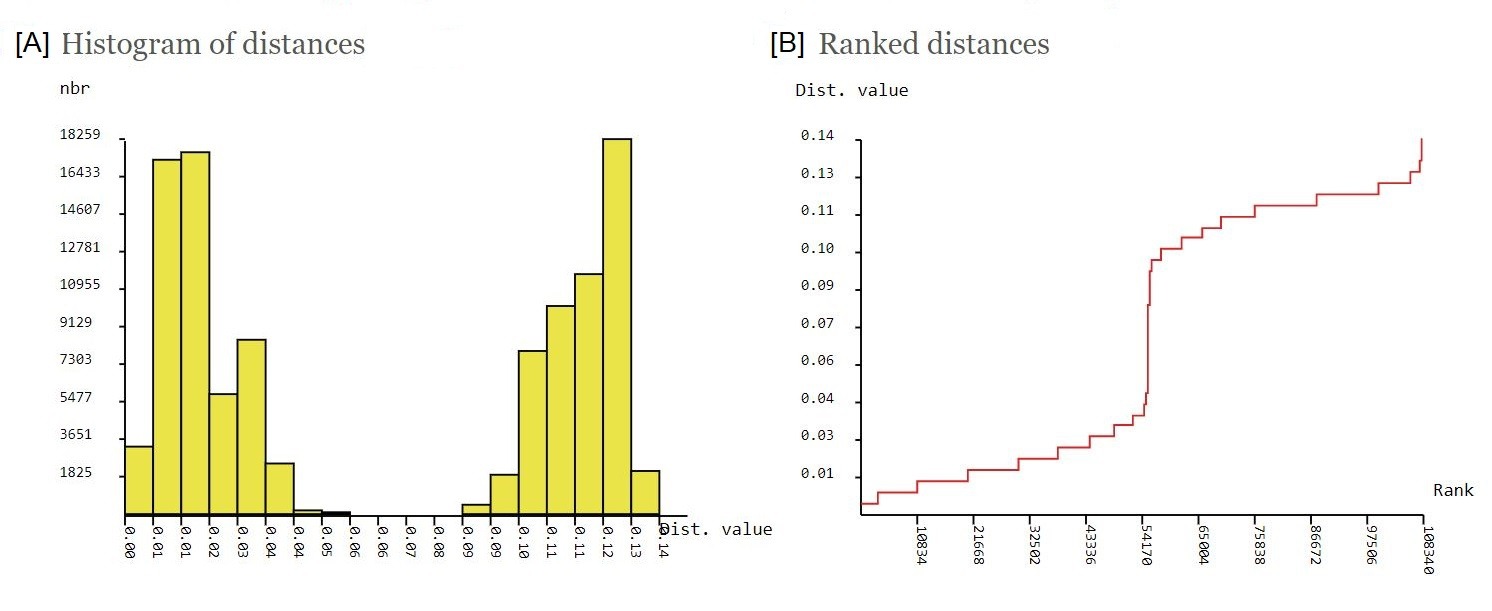

Supplement: Supplementary file 1 [file foods-09-01372-s001.zip › SUPPLEMENTARY/Figure S1_foods-943909.JPG]
